# Syntheses, Structures, and Electrochemical Properties of Metallacyclic Oxidovanadium(V) Complexes with Asymmetric Multidentate Linking Ligands

**DOI:** 10.3390/molecules29081700

**Published:** 2024-04-09

**Authors:** Kyoko Hasegawa, Masahiro Muto, Masanobu Hamada, Yasunori Yamada, Tadashi Tokii, Masayuki Koikawa

**Affiliations:** Department of Chemistry and Applied Chemistry, Faculty of Science and Engineering, Saga University, Honjo 1, Saga 840-8502, Japanyyamada@cc.saga-u.ac.jp (Y.Y.); tokiit@edu.cc.saga-u.ac.jp (T.T.)

**Keywords:** metallacycle, linking ligand, ligand design, vanadium, X-ray structures, redox chemistry

## Abstract

Trinuclear metallacyclic oxidovanadium(V) complexes, [{VO(L^3+2^R)}_3_] (**1**–**3**) with asymmetric multidentate linking ligands (H_3_L^3+2^R: R = H, Me, Br), were synthesized. The molecular structure of **1** is characterized as a tripod structure, with each V(V) ion coordinated by *ONO*-atoms from a tridentate Schiff base site and *ON*-atoms from a bidentate benzoxazole site of two respective H_3_L^3+2^H ligands. The intramolecular V⋯V distances range from 8.0683 to 8.1791 Å. Complex **4** is a mononuclear dioxidovanadium(V) complex, (Et_3_NH)[VO_2_(HL^3+2^H)]. Cyclic voltammograms of **1**−**3** in DMF revealed redox couples attributed to three single-electron transfer processes.

## 1. Introduction

The development of nanoscale molecular architectures featuring metallacycles is of significant interest in materials science and supramolecular chemistry due to their characteristic redox, magnetic, and mechanical functions, as well as their host–guest chemistry [1,2,3,4,5]. In any case, ligands with appropriate bridging abilities and directions are essential for organizing these complexes. For instance, many macrocyclic metal complexes are constructed using small anions like acetate and hydroxyl ions. In such cases, µ-acetato or µ-hydroxo bridges form small bending angles relative to the metal-to-metal direction, allowing for the preparation of macrocyclic metal arrangements of various sizes. However, since the preparation of such complexes often relies on chance, it is challenging to strategically advance scientific research. Hence, the careful design of linking ligands with multiple separate coordination sites is crucial for obtaining nano-molecular architectures and facilitating self-assembly [6,7]. Typically, using linking ligands oriented in a straight line, such as 4,4′-bipyridine or terephthalic acid, makes it easier to generate 1D, 2D, or 3D regular coordination polymers [8,9,10]. Therefore, it is essential for the coordination sites of linking ligands to not be aligned in a straight line in order to obtain discrete metallacyclic complexes.

**Scheme 1:**
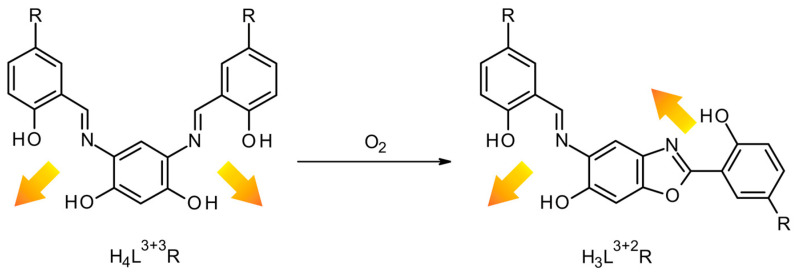
Chemical diagrams of H_4_L^3+3^R and H_3_L^3+2^R (R = H, Me, Br). Arrows indicate the coordination direction of each binding site.

In our study, we reported the synthesis of the linking ligand H_3_L^3+2^H: 2-(2-hydroxyphenyl)-6-ol-5-(salicylideneamino)benzoxazole, which has separated [3 + 2] *ONO*-tridentate and *ON*-bidentate coordination sites [11]. This ligand was obtained through the oxidation of H_4_L^3+3^H (4,6-bis[(2-hydroxybenzylidene)imino]benzene-1,3-diol) (Scheme 1), which features symmetrical [3 + 3] *ONO*-tridentate sites [12,13]. These linking ligands are expected to significantly facilitate the construction of cyclic molecules rather than coordination polymers, given the approximately 120-degree bending of the coordination directions of the separated binding sites. Indeed, the [3 + 2] asymmetric coordination sites in the H_3_L^3+2^H ligand bound to different metal ions in different styles and formed a trinuclear metallacyclic manganese(III) complex, [{Mn(L^3+2^H)(MeOH)}_3_], with a tripodal molecular structure [11]. Based on the reactivity of the ligand itself and the composition of the obtained Mn(III) complex, it is likely that this ligand readily forms complexes with redox-active metal ions in the +3 charge. Therefore, we focused on synthesizing VO^3+^ complexes, which are expected to find applications in catalysis development utilizing the high oxidation ability of V(V) [14,15,16]. Moreover, they are anticipated to serve as model complexes for metalloenzymes, contributing to our understanding of the role of vanadium ions in bioinorganic chemistry, including those present in the active sites of haloperoxidase [17,18,19] or the vanadium-containing anion amavadin, found in certain mushrooms [20].

In this study, we prepared new trinuclear metallacyclic oxidovanadium(V) complexes and a mononuclear dioxidovanadium(V) complex using these ligands. Herein, we describe the synthesis, X-ray structural characterization, and electrochemical properties of these newly obtained complexes.

## 2. Results and Discussion

The reactions of H_4_L^3+3^R·HCl (R = H, Me, Br) with vanadium(III) chloride yielded trinuclear oxidovanadium(V) complexes **1**–**3**. Elemental analysis data for all complexes indicated a 1:1 metal-to-ligand ratio. The *ν*(C=N) vibrations, which are observed at 1632~1634 cm^−1^ for the free ligands, are shifted to 1602~1617 cm^−1^ in all complexes, suggesting that each coordination site of the ligands acts as a chelate. It is presumed that each vanadium ion exists as VO^3+^, as indicated by the *ν*(V=O) vibrations observed at 967, 968, and 973 cm^−1^, respectively. The reaction of H_3_L^3+2^H with [VO(acac)_2_] (vanadium(IV) bis(acetylacetonato)oxide) yielded mononuclear dioxidovanadium(V) complex **4**. Elemental analysis data for **4** showed a 1:1 metal-to-ligand ratio. The IR spectrum of **4** exhibited two characteristic bands at 941 (weak) and 861 (strong) cm^−1^, which are assigned to the symmetric and asymmetric *ν*(O=V=O) vibration of the *cis*-VO_2_ group [21,22]. Additionally, two *ν*(C=N) vibrations similar to those of free H_3_L^3+2^H were observed at 1634 and 1615 cm^−1^. Even when using other metal sources such as VCl_3_, the yield decreases, but all isolated complexes were dioxidovanadium(V) complexes. Therefore, it is presumed that in the reaction between the oxidized ligand H_3_L^3+2^H and vanadium, the mononuclear dioxidovanadium(V) complex is preferentially formed because the oxidation of the ligand is not required. To obtain polynuclear vanadium complexes like complexes **1**−**3**, it is necessary to start the synthesis with H_4_L^3+3^R·HCl as the ligands.

### 2.1. Crystal Structures

The molecular structures of **1** and **4** were confirmed by single-crystal X-ray structure analyses. The crystallographic data and collection details for **1** and **4** are summarized in Table 1.

**Figure 1:**
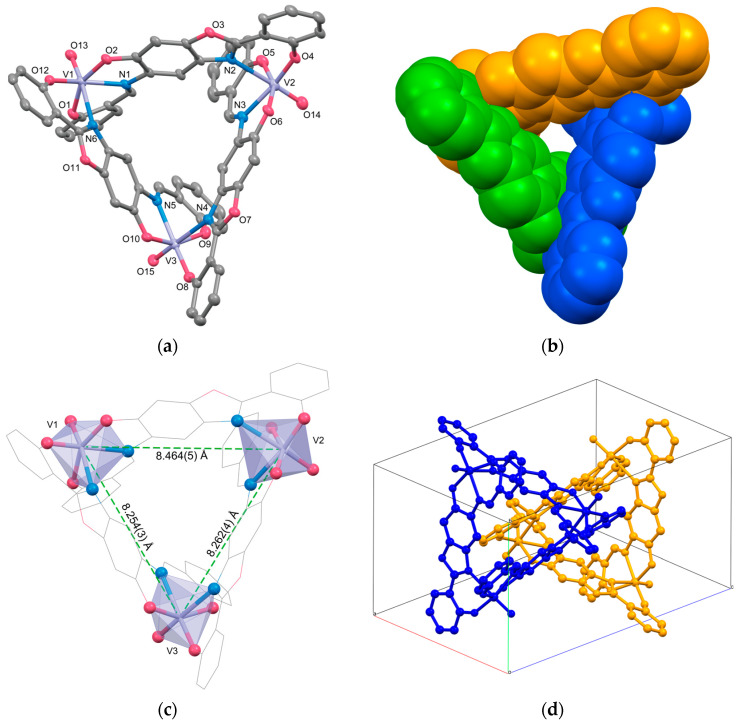
(**a**) X-Ray crystal structure of **1** showing thermal ellipsoids drawn at a 30% probability level. Hydrogen atoms have been omitted for clarity. (**b**) CPK model of **1**. Each [VO(L^3+2^H)] monomer unit is color coded. (**c**) Coordination polyhedron of V(V) with ligand framework (line drawing). Green dotted lines are guided lines for V⋯V separation. (**d**) Enantiomeric pair (blue = *x*, *y*, *z* and orange = 1 − *x*, 1 − *y*, 1 − *z*) formed by intermolecular π-π stacking interaction.

The molecular structures of **1** are illustrated in Figure 1a,b, and the selected bond lengths and angles are listed in Table 2. Complex **1** consists of three sets of ligands and oxidovanadium(V) ions, forming an overall tripod structure. It has been confirmed that the ligand H_4_L^3+3^H, used as the starting material, undergoes oxidation to H_3_L^3+2^H, similar to the previously reported manganese complex [{Mn(L^3+2^H)(MeOH)}_3_] [11]. The coordination geometry around each oxidovanadium(V) ion adopts a distorted octahedral structure, coordinated by two distinct ligands. The first donor set is the *ONO*-tridentate biding site of (L^3+2^H)^3−^. The N=C bond lengths (1.30—1.34 Å) in these tridentate sites closely resemble standard N(*sp^2^*)=C(*sp^2^*) double bonds. The sum of bond angles around N1, N3, and N5 is 359.3, 359.1, and 358.5°, respectively, suggesting that the tridentate site is bound in the Schiff base form. The second donor set consists of the *ON*-donor set from the bidentate benzoxazole site. The equatorial planes of V(V) centers are defined by the *ONO*-donors of the tridentate Schiff base site and a phenolic oxygen atom of the bidentate site, while the nitrogen atom of the benzoxazole moiety and the V=O bond form the axial axis. The bond lengths in the equatorial plane are 1.820–1.944 Å for V–O and 2.099–2.121 Å for V–N, falling within the expected range reported range reported for oxidovanadium(V) complexes with Schiff base ligands [15,21,22,23,24]. In the axial direction, the V=O bond lengths are 1.601(2), 1.609(3), and 1.606(2) Å, while the V–N_oxazole_ lengths are 2.325–2.415 Å, considerably longer due to the *trans*-influence exerted by the oxido ion as a strong π-donor. The three V(V) ions in the complex are isolated, reflected in the relatively long V⋯V distances [8.464(5), 8.254(3), and 8.262(4) Å], attributable to the characteristic ligand structure (Figure 1c).

The packing diagram of **1** is shown in Figure 1d. Enantiomer pairs loosely aggregate as dimers in the crystal lattices. In this arrangement, the phenyl rings (C36–C40) corresponding to the legs of the tripod-shaped molecule penetrate each other’s cavities. Additionally, three types of π-π stacking interactions between dimers play crucial roles in the crystal packing of the complex. Each trinuclear complex is stacked by weak π-π stacking interactions between π-conjugate systems of (L^3+2^H)^3−^ [C52…C55^k^ 3.62(2), C10…C20^l^ 3.62(2) Å, symmetry code k: 1 − *x*, −*y*, 1 − *z,* l: 1 − *x*, 1 − *y*, −*z*]. Consequently, the nearest intermolecular V⋯V distance [V2⋯V3^i^ 7.142(3) Å] is shorter than intramolecular V⋯V distances. Since only poor-quality crystals of **2** could be obtained and X-ray analysis was insufficient, the data for **2** are provided in the Supporting Information (Figure S1, Table S1). Here, we will only state that it has a similar structure to Complex **1**.

**Figure 2:**
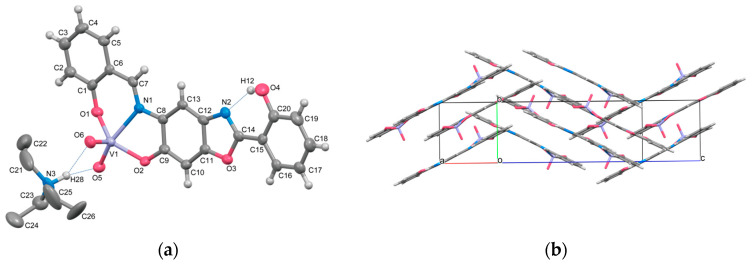
(**a**) X-Ray crystal structure of Complex **4** showing thermal ellipsoids drawn at a 30% probability level. Hydrogen atoms of ethyl groups of the triethylammonium ion have been omitted for clarity. Hydrogen bonds are drawn as dotted blue lines. (**b**) Crystal packing view of **4**. Triethylammonium ions are omitted for clarity.

The molecular structure of **4** is depicted in Figure 2a, and the selected bond lengths and angles are listed in Table 3. Complex **4** is a mononuclear dioxidovanadium(V) complex comprising the [VO_2_(HL^3+2^H)]^−^ anion and the triethylammonium cation (Et_3_NH^+^). The coordination environment of the vanadium is a distorted square pyramid with NO_4_ coordination. Two oxide ions are in a *cis*-configuration, similar to previously reported VO_2_^+^ complexes with tridentate ligands [21,22,23,24]. The V=O bond lengths are 1.642(4) and 1.634(5) Å, falling within the expected range for vanadium(V) complexes [21,22,23,24]. The V–O(phenol) bond lengths are 1.885(5) and 1.941(5) Å, and the V–N length is 2.208(5) Å. The V–N bond length is slightly longer than that of [VO(sap)(OEt)(EtOH)] (H_2_sap = *N*-(2-hydroxyphenyl)salicylideneimine) (2.114(5) Å) [25], possibly influenced by the *trans*-influence arising from the oxido ion as a strong π-donor. The central vanadium atom deviates by 0.4719(32) Å from the basal plane defined by O1, O2, O5, and N1 atoms toward the apical atom O6. The geometry index τ for five-coordination system, proposed by Addison and Rao [26], is calculated as 0.31 for the V1 environment, indicating a distorted square pyramid.

The bidentate *ON*-donor set defined by O4 and N2 does not function as a coordination site in this complex. However, this complex can be expected to act as a complex ligand capable of forming metal-assembled complexes by combining with various types of metal ions at the *ON*-site. The deviation of the mean plane defined by all atoms of the (HL^3+2^H)^2−^ ligand in **4** is 0.0476 Å, indicating good coplanarity of the ligand and an advantage for forming π-π stacking interactions. Indeed, π-π stacking interactions play important roles in the crystal packing, as shown in Figure 2b. The distance between mean planes is 3.46 Å for the (2 − *x,* 2 − *y,* 1 − *z*) unit and 3.36 Å for the (−*x,* 1 − *y,* −*z*) unit, respectively. Additionally, hydrogen bonding interactions were observed between the oxygen atoms O5, O6, and the Et_3_NH^+^ ion. The overall crystal structure of **4** closely resembles that of (Et_3_NH)[VO_2_L] (H_2_L = 4-((*E*)-(2-hydroxy-5-nitrophenylimino)methyl)benzene-1,3-diol), not only in terms of complex ion structure but also in composition and hydrogen bonding aspects [21].

### 2.2. Electrochemical Properties

**Figure 3:**
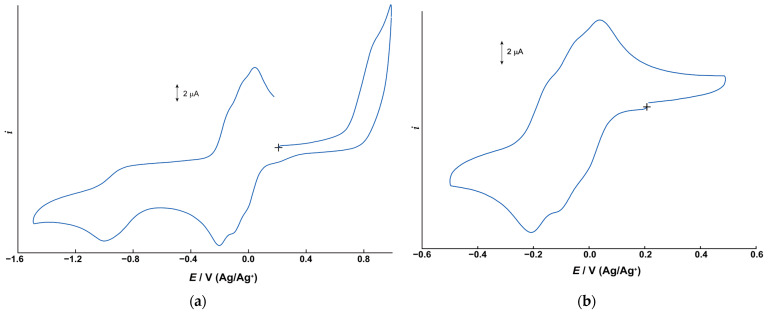
Cyclic voltammogram of **1**. 1.0 × 10^−3^ M DMF solution with 0.1 M TBAP. The cross mark indicates the starting position of the voltammogram. (**a**) Initial *E*: +0.208 V, high *E*: +0.9 V, low *E*: −1.5 V, scan speed: 100 mV/s, scan direction: positive. (**b**) Initial *E*: +0.208 V, high *E*: +0.5 V, low *E*: −0.5 V, scan speed: 100 mV/s, scan direction: negative.

The electrochemical properties of trinuclear complexes were investigated using cyclic voltammetry (CV) in *N,N*-dimethylformamide (DMF) containing tetrabutylammonium perchlorate (TBAP) (0.1 M) as the supporting electrolyte. Figure 3a,b depict the cyclic voltammograms for Complex **1**, measured within ranges from −1.5 to +0.9 V (vs. Ag/Ag^+^) (Figure 3a) and from −0.5 to +0.5 V (Figure 3b). Although somewhat indistinct, three consecutive redox couples were observed in the range of −0.2 V to 0.0 V. Since the ligand does not undergo redox reactions in this region (Figure S2), these waves are attributed to the participating redox process of the metal center. Additionally, the irreversible waves observed near +0.8 V and −1.0 V are attributed to the oxidation wave of the phenolic oxygen and the reduction wave of the azomethine in the ligand, respectively. In the voltammogram of **1**, three redox processes were roughly identified at *E*_1/2_ = −0.18, −0.09, and +0.01 V (vs. Ag/Ag^+^). These can be attributed to three one-electron transfer processes for the three vanadium centers, specifically V_3_(IV,IV,IV)/V_3_(IV,IV,V), V_3_(IV,IV,V)/V_3_(IV,V,V), and V_3_(IV,V,V)/V_3_(V,V,V) (Table 4). The peak separation values, Δ*E*_p_ = |*E*_pa_ − *E*_pc_|, for each redox pair are approximately 60–70 mV, and changing the scan rate makes it difficult to distinguish the shoulders. Therefore, these three redox processes are considered quasireversible electron transfer processes. Although oxidovanadium(V) complexes were obtained in the present studies, isolating oxidovanadium(IV) complexes is anticipated with the development of appropriate synthetic conditions. The potential differences (Δ*E*_1/2_) between the three metal-centered redox potentials exceed 100 mV. This value is considerably larger than the 28 mV expected for a purely statistical treatment in a three-electron process [27]. Generally, large bridging ligands lead to long metal-to-metal distances, resulting in small Δ*E*_1/2_ values close to the statistical case in multinuclear complexes formed by such ligands. However, in the case of the ligand H_3_L^3+2^H, the two coordination sites are connected by a π-conjugated system. Despite the vanadium ions being separated by over 8 Å in the complex structure, the enhancement of electronic repulsion effects via the π-conjugated system resulted in large Δ*E*_1/2_ values. Similar phenomena have been observed in other multinuclear complexes with long metal-to-metal distances, such as Complex **1** [28,29,30].

**Figure 4:**
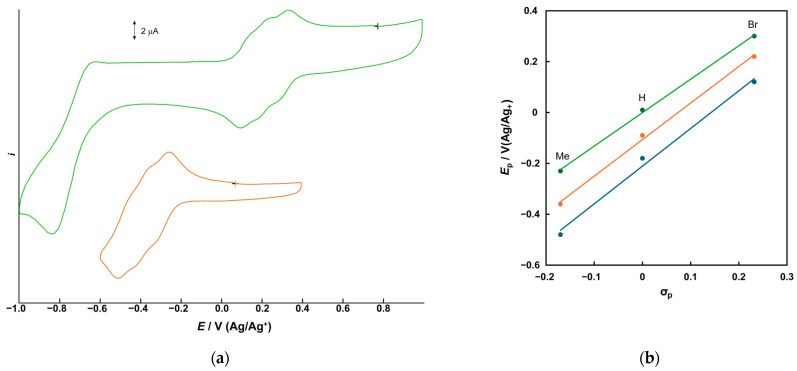
(**a**) Cyclic voltammogram of **2** (orange) and **3** (green). DMF solution (1.0 × 10^−3^ M) with 0.1 M TBAP. Scan speed: 100 mV/s. The cross mark indicates the starting position of the voltammogram. (**b**) Correlation plots between *E*_1/2_ and Hammett parameters. Green: V_3_(IV,V,V)/V_3_(V,V,V). Orange: V_3_(IV,IV,V)/V_3_(IV,V,V). Blue: V_3_(IV,IV,IV)/V_3_(IV,IV,V).

For complexes **2** and **3**, the influence of electronic properties of substituents on their redox potentials was observed (Figure 4a). Three redox couples observed for **2** were shifted by about 0.3 V to the negative side compared to those of **1**. Conversely, for Complex **3**, the redox processes were observed at approximately 0.3 V on the positive side. The electrochemical data for complexes **1**–**3** are summarized in Table 4. The three obtained *E*_1/2_ potentials were plotted against Hammett’s parameters σ_p_ for the substituents (Figure 4b). Good correlations (R^2^ = 0.992, 0.998, and 0.999) were observed, as reported in many other redox-active complexes [31,32,33].

**Figure 5:**
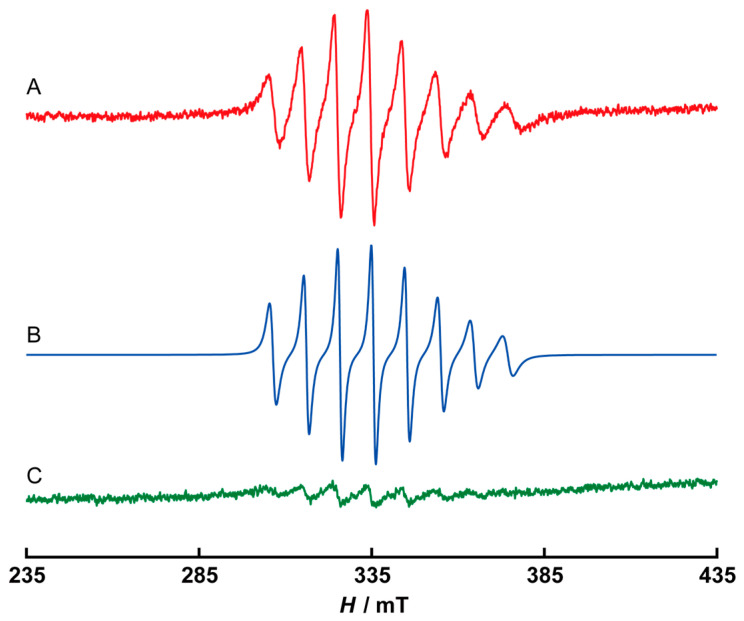
X-band solution ESR spectra of **1** at room temperature. Red line A: after reduction. Blue line B: simulated spectrum. Green line C: after oxidation of reduced solution.

To investigate the stability of the reduced state of the trinuclear complexes, controlled-potential electrolysis of Complex **1** was conducted in DMF at −0.5 V, and the ESR spectra were measured after electrolysis. The ESR spectra of the obtained solutions are shown in Figure 5. Although Complex **1** typically exhibits no ESR signals due to their *d*^0^-configration, the reduced solution exhibited ESR signals (line A) at *g* = 1.985, with a hyperfine structure originating from the nuclear spin of the vanadium nucleus (*I* = 7/2). ESR simulation (line B) was performed based on the isotropic spin Hamiltonian.

*H* = *gβHS* + Σ*A_i_SI_i_* (1)

The simulation was conducted using a Gaussian function. The half with parameter *w* for the Gaussian function was defined as *w* = *w*_1_ + *w*_2_ × |*m_I_*| + *w*_3_ × |*m_I_*|^2^, where *m_I_* is the nuclear spin quantum number. The calculation was performed using HyperFine 2.8 ESR simulation software [34]. The obtained ESR parameters are as follows: *g*_0_ = 1.983, *A*_0_ = 90.00 × 10^−4^ cm^−1^, *w*_1_ = 1.2, *w*_2_ = 0.14, *w*_3_ = 0.08. It is presumed that the oxidovanadium(IV) ion is coordinated by an *ONO*-donor set because the obtained *A*_0_ value is closer to the values for [VO(salen)] and [VO(acacen)] (H_2_acacen = *N*,*N*′-ethylenebis(acetylacetoniminate)) than that for [VO(acac)_2_] [35,36,37]. Although CV confirmed that **1** maintains its trinuclear structure in DMF, no signals comprising 15–22 lines, attributed to the total nuclear spin of the three vanadium ions or fine structure due to unpaired electrons, were observed in the ESR spectrum after electrochemical reduction. Instead, the obtained ESR signals comprised eight lines, indicating magnetically isolated vanadium ions. This suggests that the spin density of reduction species is localized on each V(IV) center after electrochemical reduction. Subsequently, electrochemical oxidation of the reduced sample was performed at +0.3 V. The intensity of the eight-lined signals noticeably diminished (line C). These results revealed that the origins of the three redox couples is the vanadium centers, with each unpaired electron localized on each vanadium ion.

## 3. Materials and Methods

### 3.1. Materials

All common reagents and solvents were purchased and used as received unless stated otherwise. Methanol was purified by distillation over magnesium turnings, while acetonitrile was dehydrated with P_2_O_5_ and distilled before use. Tetrabutylammonium perchlorate (TBAP), used as the supporting electrolyte, was recrystallized three times from a mixed solvent of dichloromethane and hexane. (Caution: TBAP is explosive and should be handled with great care!).

### 3.2. Preparation

#### 3.2.1. Ligands

The symmetric [3 + 3] ligand, H_4_L^3+3^H·HCl, and the asymmetric [3 + 2] ligand, H_3_L^3+2^H, were obtained using methods described in [11]. The other ligands, H_4_L^3+3^Br·HCl and H_4_L^3+3^Me·HCl, were synthesized similarly by employing 5-bromo- and 5-methylsalicylaldehyde. H_4_L^3+3^Br·HCl·2H_2_O: Yield 1.41 g, 61%. C_20_H_19_Br_2_ClN_2_O_6_ (578.64): calcd. C 41.51, H 3.31 N 4.84; found C 41.27, H 3.23, N 4.91%. IR(KBr): $\tilde{\nu }\;$= 3338 and 3039 (OH), 1634 (C=N), 827 (phenyl) cm^−1^. ^1^H NMR (400 MHz; DMSO; TMS) *δ* = 9.08(s, 2H), 7.80(s, 1H), 7.10(s, 2H), 7.22(d, 2H), 6.88(d, 2H), 6.74(s, 1H), 2.25(s, 6H) ppm. H_4_L^3+3^Me·HCl·2.5H_2_O: Yield 0.860 g, 94%. C_22_H_26_ClN_2_O_6.5_ (457.9): calcd. C 57.71, H 5.72 N 6.12; found C 57.70, H 5.54, N 6.12%. IR(KBr): $\tilde{\nu }$ = 3370 and 3075 (OH), 1632 (C=N), 827 (phenyl) cm^−1^. ^1^H NMR (300 MHz; DMSO; TMS) *δ* = 9.02(s, 2H), 7.78(s, 2H), 7.62(s, 1H), 7.50(d, 2H), 6.92(d, 2H), 6.66(s, 1H) ppm.

#### 3.2.2. [{. VO(L^3+2^R)}_3_] (R = H: **1**, Me: **2**, Br: **3**)

Triethylamine (0.03 g, 0.3 mmol) was added dropwise to a mixture of vanadium(III) chloride (0.016 g, 0.1 mmol) and H_4_L^3+3^H·HCl (0.042 g, 0.1 mmol) in 15 mL of acetonitrile with stirring. Subsequently, 3 mL of DMF was added to the resulting dark brown solution, followed by filtration. The filtrate was allowed to stand for one day at room temperature. Single crystals of **1** suitable for X-ray crystallography were obtained from the filtrate. Complexes **2** and **3** were obtained using the same synthetic method as **1**, employing H_4_L^3+3^Me·HCl and H_4_L^3+3^Br·HCl as ligands, respectively. **1**·0.5MeCN·2H_2_O: Yield 55.9%. C_61_H_38.5_N_6.5_O_17_V_3_ (1287.33): calcd. C 56.91, H 3.01, N 7.07, V 11.87; found C 56.88, H 3.11, N 6.98, V 11.97%. IR (KBr) [cm^−1^]: $\tilde{\nu }\;$= 1602–1617 (C=N), 967 (V=O). **2**·MeCN·H_2_O: Yield 0.013 g, 28.4%. C_68_H_50_N_7_O_16_V_3_ (1374.00): calcd. C 59.44, H 3.67, N 7.14, V 11.12; found C 59.02, H 3.73, N 6.82, V 11.53%. IR (KBr) [cm^−1^]: $\tilde{\nu }\;$= 1604–1616 (C=N), 968 (V=O). **3**·1.5DMF·H_2_O: Yield 22.9%. C_64.5_H_39.5_Br_6_N_7.5_O_17.5_V_3_ (1831.79): calcd. C 42.29, H 2.17, N 5.73, V 8.34; found C 42.59, H 2.04, N 5.25, V 7.89% IR (KBr) [cm^−1^]: $\tilde{\nu }\;$= 1608–1615 (C=N), 973 (V=O).

#### 3.2.3. (. Et_3_NH)[(VO_2_)(HL^3+2^H)] (**4**)

Triethylamine (0.10 g, 1.0 mmol) was added dropwise to a mixture of vanadium(IV) bis(acetylacetonato)oxide (0.054 g, 0.2 mmol) and H_3_L^3+2^H (0.084g, 0.2 mmol) in dichloromethane (15 mL) with stirring. The resulting dark brown solution was filtered, and the filtrate was allowed to stand for one week at room temperature. Orange microcrystals were filtered off, washed with dry acetonitrile, and dried in vacuo. Single crystals suitable for X-ray crystallography were obtained from the filtrate after three months. **4**·0.2H_2_O: Yield 0.015 g, 12.9%. C_26_H_28.4_N_3_O_6.2_V (533.06): calcd. C 58.58, H 5.37, N 7.88, V 9.56; found C 58.18, H 5.28, N 7.81, V 9.40%. IR (KBr): $\tilde{\nu }\;$= 1615, 1634 (C=N), 861, 941 (V=O) cm^−1^.

### 3.3. Physical Measurements

Elemental analyses for C, H, and N were conducted at the Elemental analysis Service Center, Kyushu University. Analyses of vanadium were carried out using a titrimetric method [38]. Infrared spectra were recorded on a PerkinElmer Spectrum 2000 FT-IR Spectrometer (PerkinElmer, Inc., Waltham, MA, USA) on KBr disks. The IR spectra for the newly synthesized compounds are presented in Figures S3–S8. Nuclear magnetic resonance spectra were recorded on a Varian 400MHz NMR System (Agilent Technologies, Santa Clara, CA, USA) and a JNM-AL300 Spectrometer (JEOL, Ltd., Tokyo, Japan) in DMSO-*d*_6_ using tetramethylsilane as the internal standard. The NMR spectra for the newly obtained ligands are shown in Figure S9. The cyclic voltammogram (CV) was obtained on a BAS CV-27 Electrochemical Voltammetric Analyzer (BAS, Inc., Tokyo, Japan). The measurements of CV were carried out in DMF containing 0.1 M TBAP as the supporting electrolyte. A three-electrode cell was used, equipped with a glassy carbon (ϕ = 3 mm) working electrode, a platinum wire counter electrode, and an Ag/Ag^+^ electrode as the reference. X-band ESR spectra were recorded on a JEOL JES-TE300 ESR Spectrometer (JEOL, Ltd., Tokyo, Japan) at room temperature.

### 3.4. X-ray Crystallography

Diffraction data for **1** were measured on a Rigaku Vari-Max Saturn CCD 724 diffractometer (Rigaku Corp, Tokyo, Japan) with graphite monochromated Mo Ka radiation (λ = 0.71069 Å) at the Analytical Research Center for Experimental Sciences, Saga University. Data were collected and processed using CrystalClear [39]. The crystal was maintained at 113 K during data collection. Absorption correction was applied by the multi-scan method. The structures were solved by direct methods (ShelXT) and expanded using Fourier techniques [40]. Non-hydrogen atoms were refined anisotropically, while hydrogen atoms were placed geometrically in calculated positions and refined using a riding model. The crystal contained a large number of highly disordered solvent molecules, making it difficult to determine their positions. Therefore, solvent masks were calculated for the disordered solvent molecules by PLATON/SQUEEZE [41]. For **1**, 214 electrons were found in a volume of 2919 Å^3^ in 1 void per unit cell. These are consistent with the presence of 6[H_2_O] and 8[CH_3_OH] per molecular formula for **1**. The final cycle of full-matrix least-squares refinement on *F*^2^ using ShelXL [42] was conducted based on observed reflections and variable parameters, resulting in convergence with unweighted and weighted agreement factors of *R* and *R_w_*. Olex2 [43] served as an interface to the ShelX program package. The molecular structures were drawn using Mercury [44].

The diffraction data of **2** and **4** were collected using a Rigaku AFC5S automated four-circle diffractometer (Rigaku Corp, Tokyo, Japan) with graphite-monochromated Mo*K*α (λ = 0.71069 Å) radiation. Data collection was performed at room temperature using either the ω scan technique or the ω-2θ scan technique. Weak reflections (*I* < 10σ(*I*)) were rescanned (up to a maximum of 5 rescans) to ensure reliable counting statistics, with stationary background counts recorded on each side of the reflection. The peak counting time ratio was maintained at 2:1, and the intensities of three representative reflections were measured after every 150 reflections. Empirical absorption corrections, based on azimuthal scans of several reflections, were applied. The structures were solved using direct methods (SIR92) [45] and expanded using Fourier techniques. All hydrogen atoms were located on the calculated positions and refined using the riding model. The PLATON/SQUEEZE program was applied to **2** for the calculation of the disordered solvent contribution. For **2**, 602 electrons were found in a volume of 3058 in 1 void per unit cell. The final cycle of full-matrix least-squares refinement on *F^2^* using SHELXL-2016 [40] was conducted based on observed reflections and variable parameters, resulting in convergence with unweighted and weighted agreement factors of *R* and *R_w_*. All calculations were carried out using the Rigaku CrystalStructure crystallographic software package version 4.3 [46], employing SHELXL-2016. The crystallographic data and collection details for **1** and **4** are summarized in Table 1, while the data for **2** are compiled in Table S1.

## 4. Conclusions

It has been demonstrated that asymmetric linking ligands, H_3_L^3+2^H, H_3_L^3+2^Me, and H_3_L^3+2^Br, facilitate the formation of metallacyclic trinuclear oxidovanadium(V) complexes **1**−**3**, described as [{VO(L^3+2^R)}_3_]. In these complexes, the vanadium atoms adopt six-coordinate geometries, and the molecular structures are characterized as tripodal pyramids with small cavities. The mononuclear complex (Et_3_NH)[VO_2_(HL^3+2^H)] (**4**) was obtained through the reaction of [VO(acac)_2_] with H_3_L^3+2^H, featuring a square pyramidal dioxidovanadium(V) configuration. The trinuclear complexes exhibit three consecutive redox waves, and a notable correlation has been observed between the redox potentials and Hammett parameters. The result suggests that the present ligands offer new applications for obtaining redox-active trinuclear complexes and advancing self-assembling chemistry.

## Figures and Tables

**Table 1:** Crystallographic data and refinement parameters.

	1	4
Empirical formula	C_60_H_33_N_6_O_15_V_3_	C_26_H_28_N_3_O_6_V
Formula weight	1230.74	529.47
Temperature/K	113	301
Crystal system	triclinic	Monoclinic
Space group	*P* $\bar{1}$	*P*2_1_*/n*
*a*/Å	12.465(5)	11.978(3)
*b*/Å	16.244(6)	7.5880(14)
*c*/Å	18.285(8)	28.042(5)
*α/*°	102.99(3)	90
*β/*°	98.16(3)	101.705(18)
*γ*/°	108.59(3)	90
*V*/Å^3^	3327(3)	2495.7(8)
*Z*	2	4
*D*_calc_/g cm^−3^	1.229	1.409
μ(MoKα)/mm^−1^	0.476	0.444
*F*(0 0 0)	1248.0	1104.0
Crystal dimensions/mm^3^	0.22 × 0.14 × 0.08	0.40 × 0.20 × 0.10
Radiation	MoKα (λ = 0.71075)	MoKα (λ = 0.71075)
2*θ* range for data collection/°	6.1 to 54.838	6.0 to 55.0
No. of reflections collected	26,785	5577
No. of independent reflections	14,423	5478
Data/Restraints/Params	144,23/0/758	5478/0/329
Goodness of fit indicator	0.949	0.947
*R* indices [*I* > 2.00σ(*I*)]	*R*_1_ = 0.0568	*R*_1_ = 0.0790
	*wR*_2_ = 0.1509	*wR*_2_ = 0.2160
*R* indices (all data)	*R*_1_ = 0.0852	*R*_1_ = 0.2748
	*wR*_2_ = 0.1766	*wR*_2_ = 0.3181
Largest diff. peak, hole/e Å^−3^	0.41, −0.61	0.64, −0.60
CCDC deposition number	2,338,377	2,338,378

**Table 2:** Selected bond distances and angles of **1**.

Bond	Distance/Å	Angle	Angle/°
V–O1	1.897(2)	O1–V1–O2	154.33(9)
V1–O2	1.944(2)	O12–V1–N1	162.90(10)
V1–O12	1.847(3)	O13–V1–N6	172.12(10)
V1–O13	1.601(2)	O12–V1–N6	79.98(9)
V1–N1	2.121(3)	O1–V1–N1	83.27(10)
V1–N6	2.355(3)	O2–V1–N1	77.24(10)
V2–O4	1.820(2)	O5–V2–O6	156.00(10)
V2–O5	1.862(2)	O4–V2–N3	164.98(11)
V2–O6	1.941(2)	O14–V2–N2	177.48(10)
V2–O14	1.609(3)	O4–V2–N2	81.01(10)
V2–N2	2.436(3)	O5–V2–N3	85.17(10)
V2–N3	2.110(3)	O6–V2–N3	78.29(10)
V3–O8	1.838(2)	O9–V3–O10	154.56(9)
V3–O9	1.893(3)	O8–V3–N5	162.18(11)
V3–O10	1.943(2)	O15–V3–N4	174.62(12)
V3–O15	1.606(2)	O8–V3–N4	80.91(10)
V3–N4	2.312(3)	O9–V3–N5	83.70(10)
V3–N5	2.099(3)	O10–V3–N5	77.88(10)
V1⋯V2	8.464(5)		
V1⋯V3	8.254(3)		
V2⋯V3	8.262(4)		

**Table 3:** Selected bond distances and angles of **4**.

Bond	Distance/Å	Angle	Angle/°
V1–O1	1.884(6)	O1–V1–O2	140.9(2)
V1–O2	1.941(6)	O1–V1–O5	95.8(2)
V1–O5	1.644(5)	O1–V1–O6	105.2(3)
V1–O6	1.632(6)	O1–V1–N1	81.6(2)
V1–N1	2.207(6)	O2–V1–O5	93.6(2)
		O2–V1–O6	108.5(3)
O4–H12⋯N2	2.660(9)	O2–V1–N1	75.5(2)
N3–H28⋯O5	2.900(9)	O5–V1–O6	105.8(3)
N3–H28⋯O6	2.983(9)	O5–V1–N1	157.3(3)
		O6–V1–N1	96.6(2)

**Table 4:** Electrochemical data of metal-centered redox process for **1**–**3**.

Complex	V_3_(IV,IV,IV)/V_3_(IV,IV,V) *			V_3_(IV,IV,V)/V_3_(IV,V,V) *			V_3_(IV,V,V)/V_3_(V,V,V) *		
	*E*_pc_	*E*_pa_	*E*_1/2_	*E*_pc_	*E*_pa_	*E*_1/2_	*E*_pc_	*E*_pa_	*E*_1/2_
**1**	−0.208	−0.15 **	−0.18	−0.12 **	−0.06 **	−0.09	−0.02 **	+0.038	+0.01
**2**	−0.512	−0.44 **	−0.48	−0.39 **	−0.32 **	−0.36	−0.20 **	−0.262	−0.23
**3**	+0.091	+0.15 **	+0.12	+0.18 **	+0.25 **	+0.22	+0.27 **	+0.333	+0.30

* vs. Ag/Ag^+^; ** determined by numerical differentiation.

## Data Availability

The data that support the findings of this study are available from the author upon reasonable request. The data are not publicly available due to privacy reasons.

## Supplementary Materials

The following supporting information can be downloaded at: https://www.mdpi.com/article/10.3390/molecules29081700/s1: Figure S1: X-ray crystal structure of **2**; Figure S2: Cyclic voltammograms of the ligands; Figure S3: IR spectrum of H_4_L^3+3^Me·HCl; Figure S4: IR spectrum of H_4_L^3+3^Br ·HCl; Figure S5: IR spectrum of **1**; Figure S6: IR spectrum of **2**; Figure S7: IR spectrum of **3**; Figure S8: IR spectrum of **4**; Figure S9: ^1^H-NMR Spectra of H_4_L^3+3^Me∙HCl and H_4_L^3+3^Br∙HCl in *d*_6_-DMSO; Table S1: Crystal data for **2.**
