# Supplementary material for: Syntheses, Structures, and Electrochemical Properties of Metallacyclic Oxidovanadium(V) Complexes with Asymmetric Multidentate Linking Ligands
Source: Molecules. 2024 Apr 9;29(8):1700. doi: 10.3390/molecules29081700 (PMC11052122; doi:10.3390/molecules29081700)

Supplementary Materials

Syntheses, Structures, and Electrochemical Properties of Metallacyclic Oxovanadium(V) Complexes with Asymmetric Multidentate Linking Ligands

Kyoko Hasegawa, Masahiro Muto, Masanobu Hamada, Yasunori Yamada, Tadashi Tokii, and Masayuki Koikawa*

Department of Chemistry and Applied Chemistry, Faculty of Science and Engineering, Saga University, Honjo 1, Saga 840-8502, Japan; koikawa@cc.saga-u.ac.jp

* Correspondence: koikawa@cc.saga-u.ac.jp

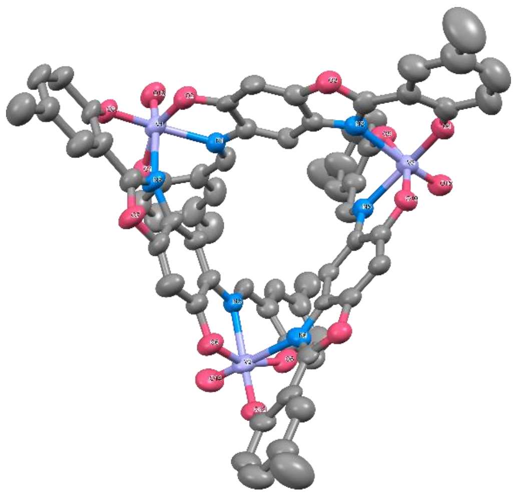

Figure S1. X-ray crystal structure of 2.

Table S1 Crystal data and structure refinement for 2.

Empirical formula	$C_{70}H_{48}N_8O_{19}V_3$	Formula weight	1458.02
Temperature / K	296		
Crystal system	triclinic	Space group	$P\bar{1}$
a / Å	15.307(5)	α / °	87.11(2)
b / Å	15.582(5)	β / °	69.06(2)
c / Å	18.458(5)	γ / °	79.74(3)
Volume / Å^3	4045(2)	Z	2
ρ_{calc}g / cm^3	1.197	Crystal size/mm^3	0.9 × 0.3 × 0.1
μ / mm^{-1}	4.056	F(000)	1490.0
Radiation	MoKα (λ = 0.71069)	2Θ range for data collection/°	6.25 to 54.974
Reflections collected	11453	Independent reflections	10895 (R_{int} = 0.0983)
Data/restraints/parameters	10895/0/863	Goodness-of-fit on F^2	0.927
Final R indexes [I>=2σ (I)]	R_1 = 0.0858<br>wR_2 = 0.2336	Final R indexes [all data]	R_1 = 0.2692<br>wR_2 = 0.3156
Largest diff. peak/hole / e Å^{-3}	0.73/−0.35		

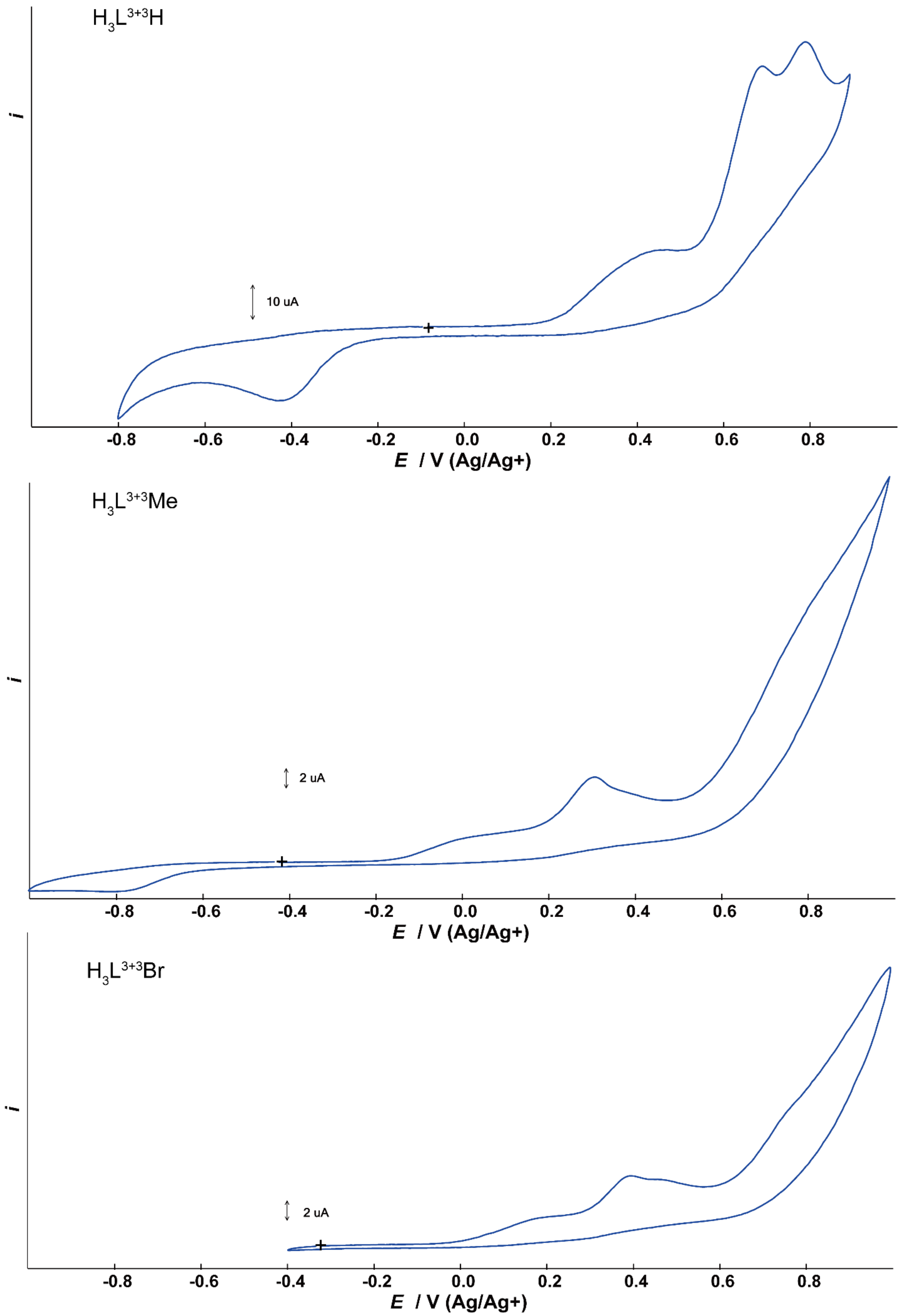

Figure S2. Cyclic voltammograms for the ligands.

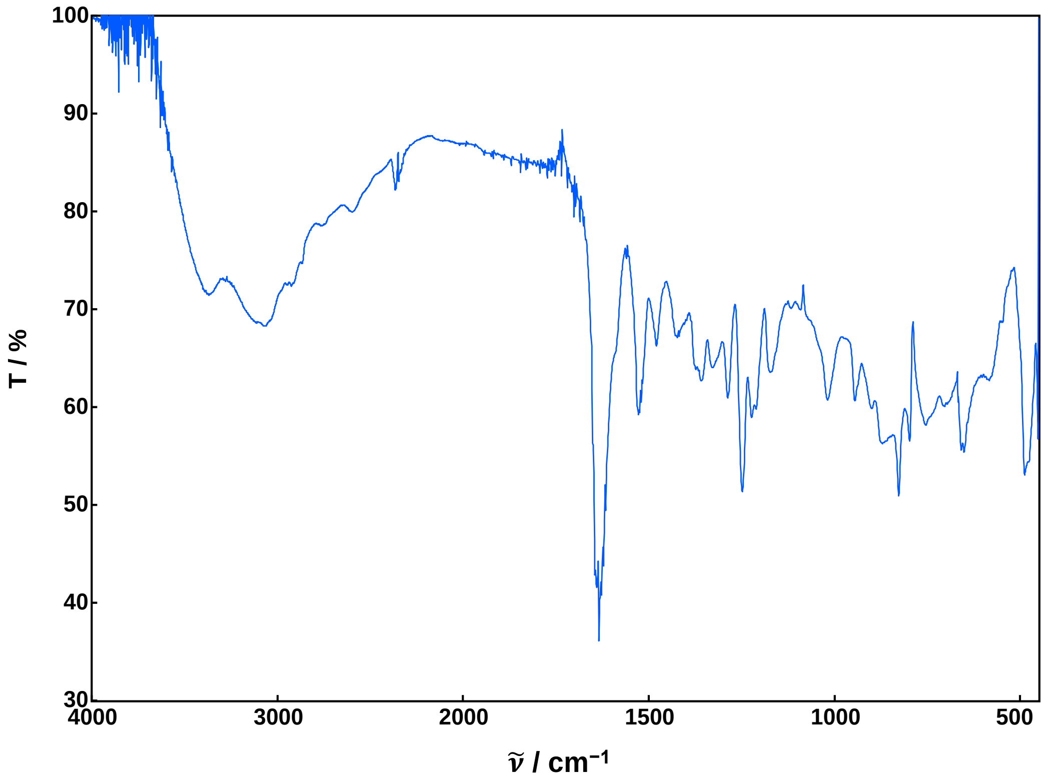

Figure S3. IR spectrum of $H_4L^{3+3}Me\cdot HCl$.

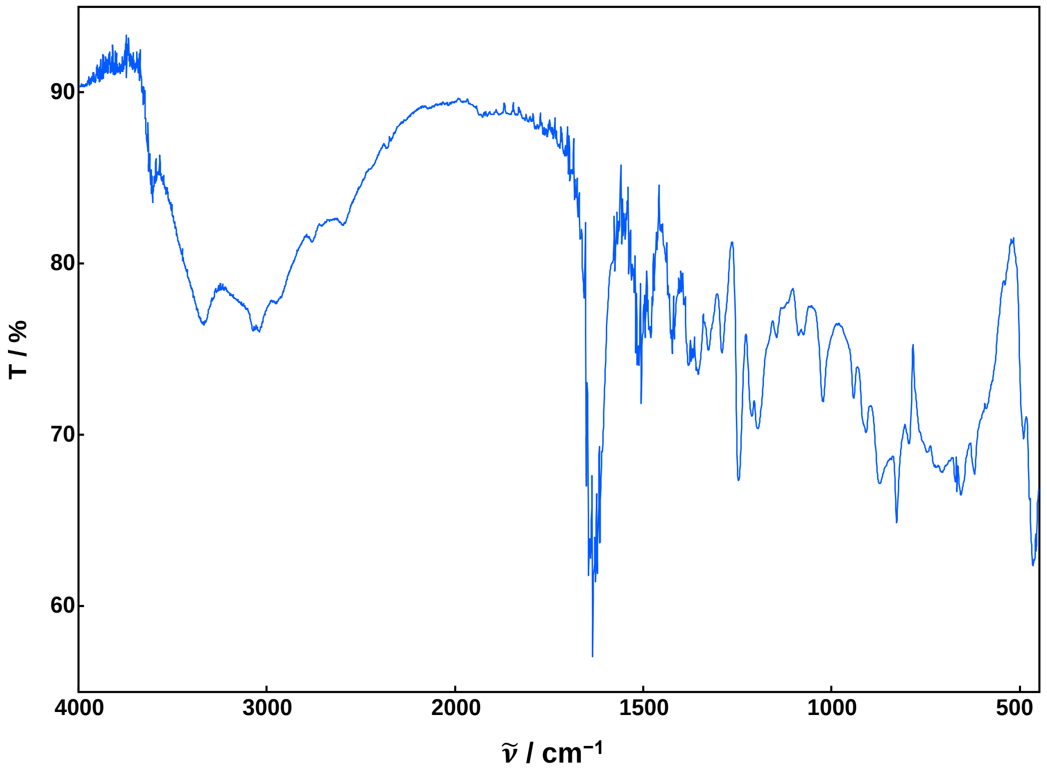

Figure S4. IR spectrum of $H_4L^{3+3}Br\cdot HCl$.

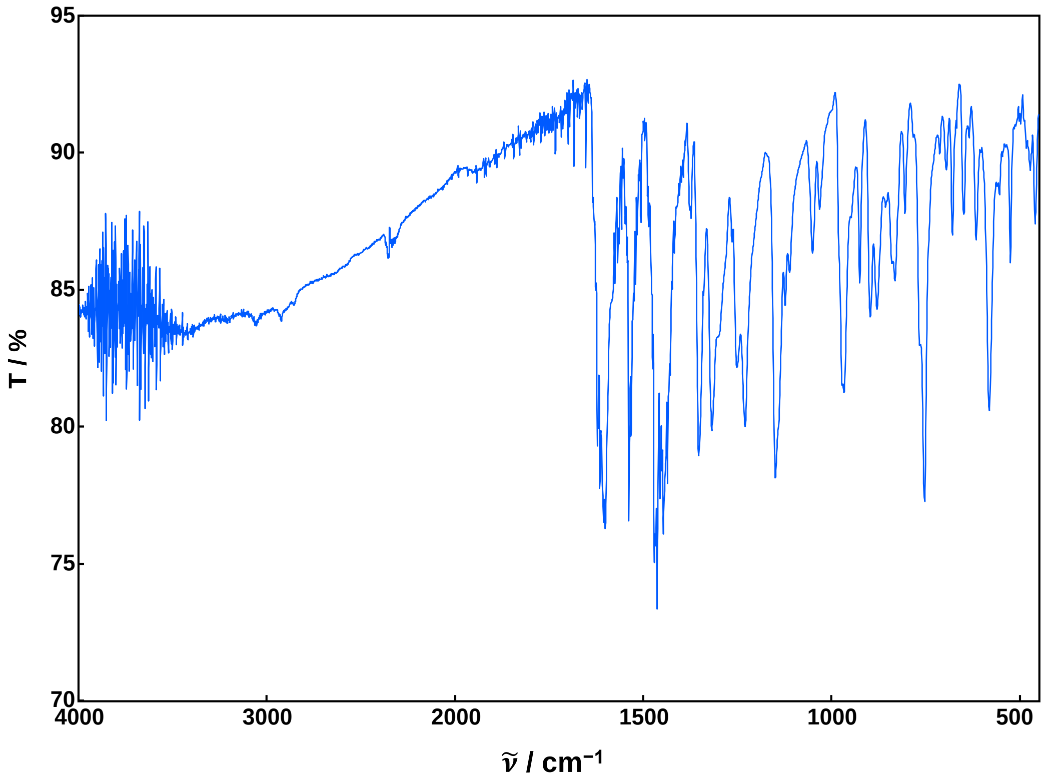

Figure S5. IR spectrum of **1**.

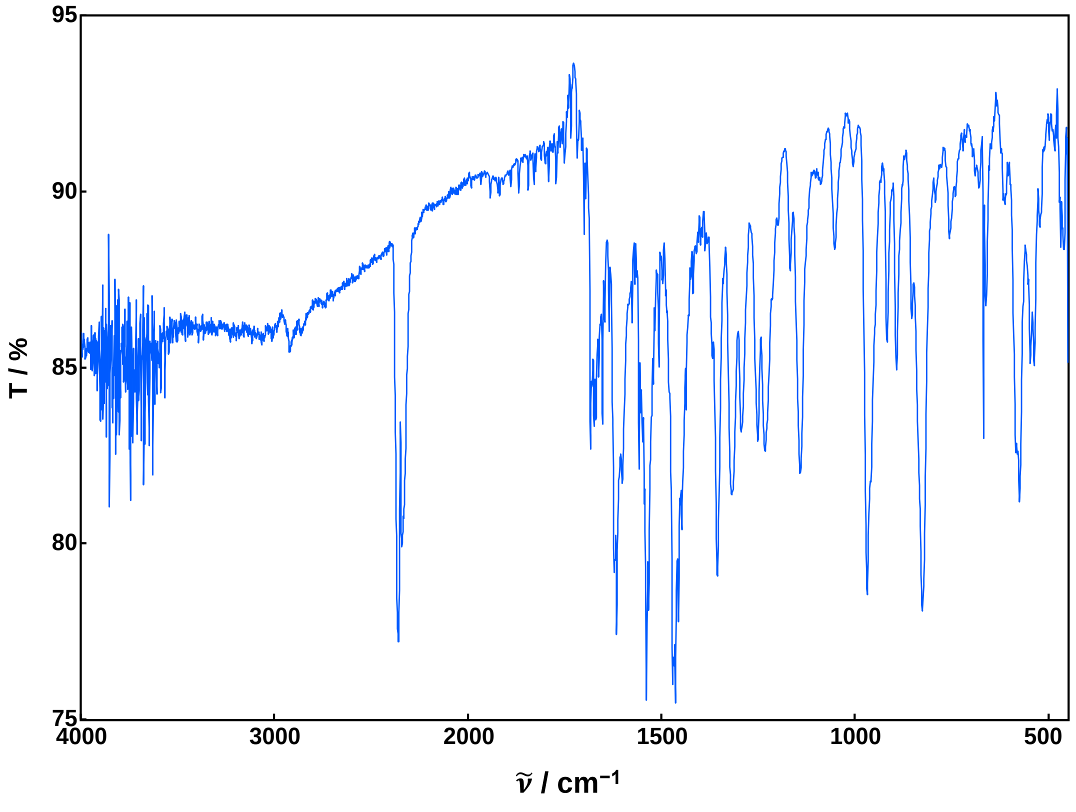

Figure S6. IR spectrum of **2**.

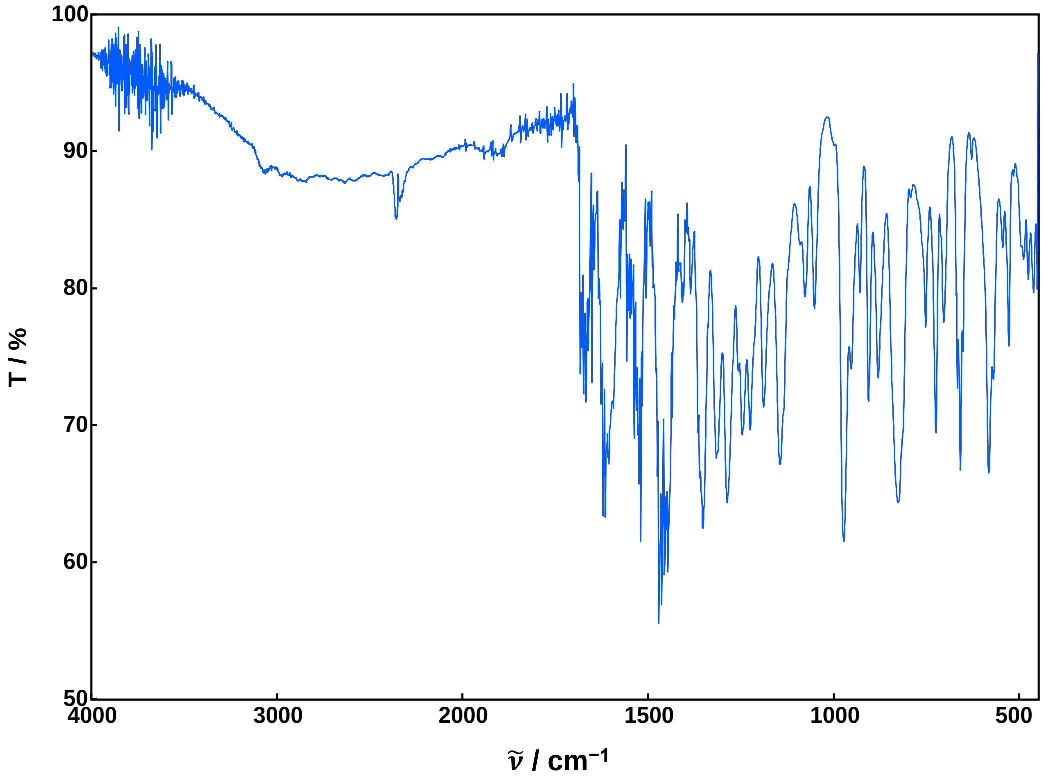

Figure S7. IR spectrum of **3**.

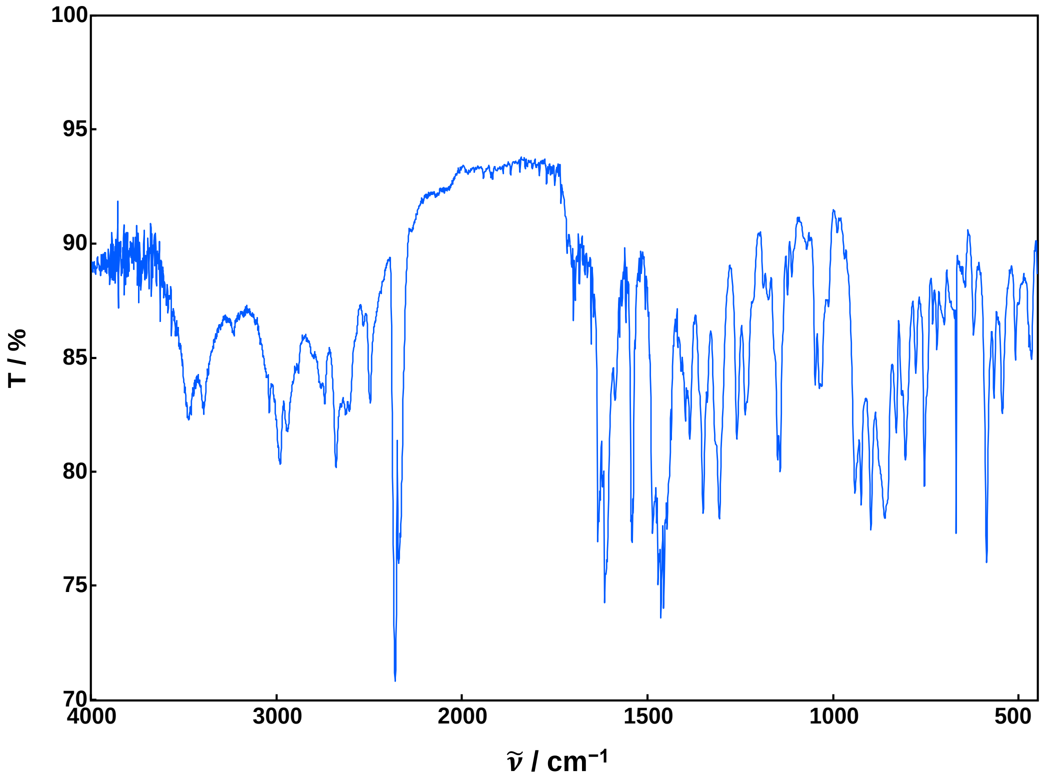

Figure S8. IR spectrum of **4**.

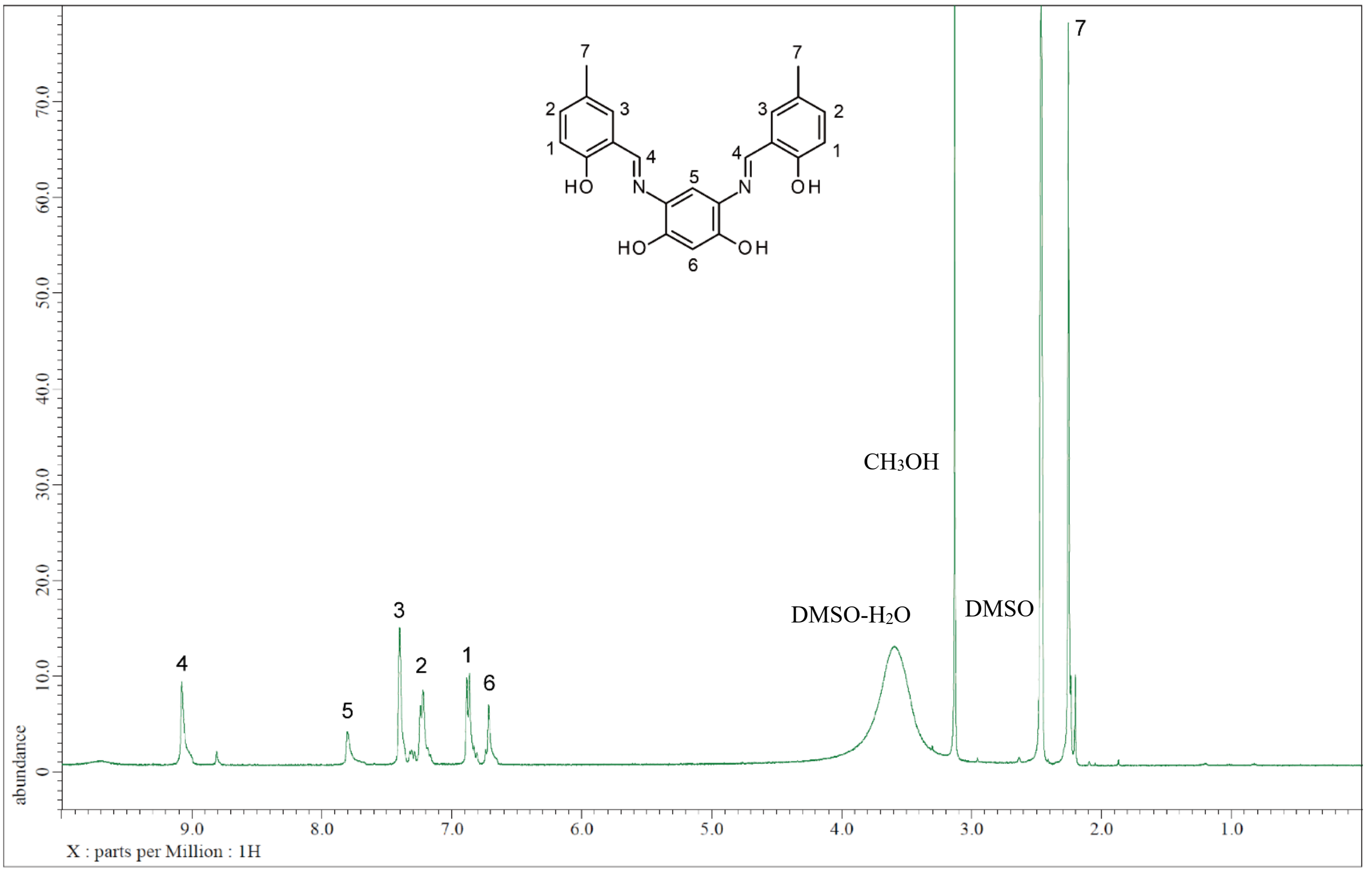

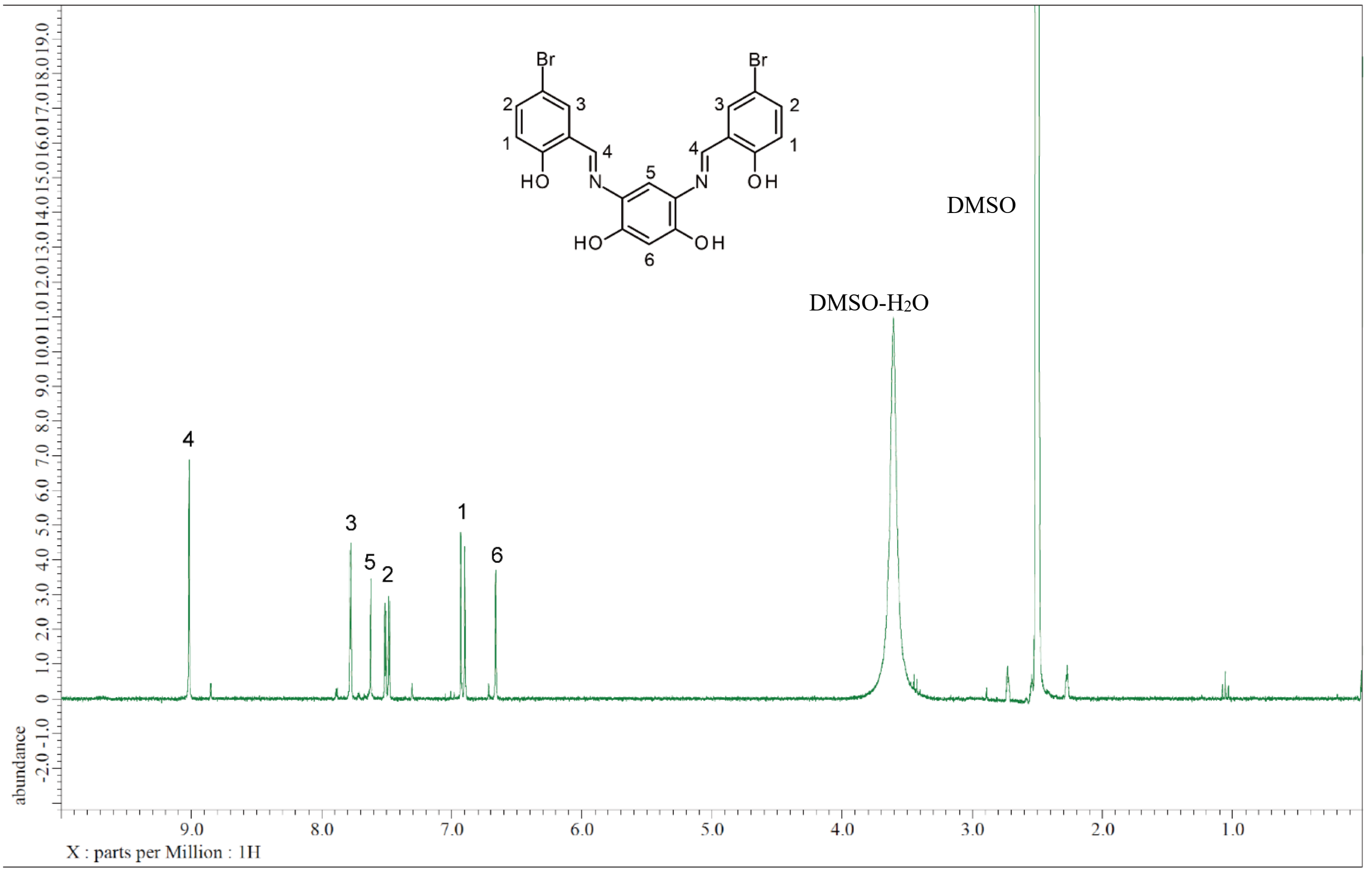

Figure S9. ^{1}H-NMR Spectra of H_4L^{3+3}Me·HCl and H_4L^{3+3}Br·HCl in d_6-DMSO.

Complex 1

checkCIF/PLATON report

Structure factors have been supplied for datablock(s) VO3L5-H3

THIS REPORT IS FOR GUIDANCE ONLY. IF USED AS PART OF A REVIEW PROCEDURE FOR PUBLICATION, IT SHOULD NOT REPLACE THE EXPERTISE OF AN EXPERIENCED CRYSTALLOGRAPHIC REFEREE.

No syntax errors found.   CIF dictionary   Interpreting this report

Datablock: VO3L5-H3

Bond precision: C-C = 0.0050 A   Wavelength=0.71075

Cell:	a=12.465(5)	b=16.244(6)	c=18.285(8)
	alpha=102.99(3)	beta=98.16(3)	gamma=108.59(3)
Temperature:	113 K		

	Calculated	Reported
Volume	3327(3)	3327(2)
Space group	P -1	P -1
Hall group	-P 1	-P 1
Moiety formula	C60 H33 N6 O15 V3 [+ solvent]	C60 H33 N6 O15 V3, [+ solventS]
Sum formula	C60 H33 N6 O15 V3 [+ solvent]	C60 H33 N6 O15 V3
Mr	1230.75	1230.74
Dx,g cm-3	1.229	1.229
Z	2	2
Mu (mm-1)	0.476	0.476
F000	1248.0	1248.0
F000’	1250.39	
h,k,lmax	16,21,23	16,20,23
Nref	15151	14423
Tmin,Tmax	0.923,0.963	0.868,1.000
Tmin’	0.901	

Correction method= # Reported T Limits: Tmin=0.868 Tmax=1.000
AbsCorr = MULTI-SCAN

Data completeness= 0.952   Theta(max)= 27.419

R(reflections)= 0.0568(8693) wR2(reflections)= 0.1766(14423)

S = 0.949 Npar= 758

The following ALERTS were generated. Each ALERT has the format
test-name_ALERT_alert-type_alert-level.
Click on the hyperlinks for more details of the test.

Alert level B

```
PLAT910_ALERT_3_B Missing # of FCF Reflection(s) Below Theta(Min).         22 Note
                1  0  0,  -1  1  0,   0  1  0,   1  1  0,  -1  2  0,   0  2  0,
                0 -2  1,   1 -2  1,  -1 -1  1,   0 -1  1,   1 -1  1,  -1  0  1,
                0  0  1,   1  0  1,  -1  1  1,   0  1  1,  -1 -1  2,   0 -1  2,
                1 -1  2,  -1  0  2,   0  0  2,  -1  1  2,
```

Author Response: Low-angle reflections rejected because of beam stop and high background scattering.

Alert level C

```
ABSTY02_ALERT_1_C  An _exptl_absorpt_correction_type has been given without
            a literature citation. This should be contained in the
            _exptl_absorpt_process_details field.
            Absorption correction given as multi-scan
CRYSC01_ALERT_1_C The word below has not been recognised as a standard
            identifier.
            dull
CRYSC01_ALERT_1_C The word below has not been recognised as a standard
            identifier.
            greenish
PLAT029_ALERT_3_C _diffrn_measured_fraction_theta_full value Low .      0.978 Why?
PLAT230_ALERT_2_C Hirshfeld Test Diff for    C59      --C60      .        7.0 s.u.
PLAT911_ALERT_3_C Missing FCF Refl Between Thmin & STh/L=    0.600        240 Report
                2  1  0,  -3  4  0,   4  4  0,   3 -6  1,   3 -4  1,  -2 -3  1,
               -1 -3  1,  -3 -2  1,  -1 -2  1,  -3 -1  1,  -2 -1  1,   3  0  1,
                5  0  1,   0  2  1,   0  3  1,   5 -1  2,  -1  4  2,   0 -4  3,
               -2  0  3,  -1  0  3,  -2  6  3,   0 -2  4,  -4 -1  4,  -1 -2  5,
                0 -2  5,   8 -2  5,   6 -4  6,  -8  0  6,  -3  3  6,  -6 11  6,
                1 -4  8, -12  1  8, -13  1 10, -14  4 10, -14  5 10, -13  1 11,
              -12  2 11, -13  4 11, -13  1 12, -13  2 12, -12  2 12, -13  3 12,
              -12  3 12, -13  4 12, -13  5 12, -13  6 12, -13  1 13, -13  2 13,
              -12  2 13, -13  3 13, -12  3 13, -11  3 13, -13  4 13, -12  4 13,
              -13  5 13, -12  5 13, -11 -1 14, -12  1 14, -12  2 14, -11  2 14,
              -12  3 14, -11  3 14, -10  3 14, -12  4 14, -11  4 14, -12  5 14,
              -11  5 14, -12  6 14, -12  7 14,  -1 -7 15, -12  0 15, -12  1 15,
              -11  1 15, -10  1 15, -12  2 15, -11  2 15, -10  2 15,  -9  2 15,
              -12  3 15, -11  3 15, -10  3 15,  -9  3 15, -12  4 15, -11  4 15,
              -10  4 15, -11  5 15, -11  6 15, -11  7 15, -11  0 16, -10  0 16,
              -11  1 16, -10  1 16,  -9  1 16,  -8  1 16,  -7  1 16, -11  2 16,
PLAT913_ALERT_3_C Missing # of Very Strong Reflections in FCF ....          6 Note
               -3 -2  1,  -3 -1  1,  -1 -1  1,   0 -1  1,  -1  4  2,   0 -2  5,
```

Alert level G

```
PLAT042_ALERT_1_G Calc. and Reported MoietyFormula Strings  Differ     Please Check
              Calc: C60 H33 N6 O15 V3
              Rep.: C60 H33 N6 O15 V3,
PLAT154_ALERT_1_G The s.u.'s on the Cell Angles are Equal ..(Note)       0.03 Degree
PLAT232_ALERT_2_G Hirshfeld Test Diff (M-X)  V3       --O10      .        5.7 s.u.
PLAT398_ALERT_2_G Deviating  C-O-C   Angle From 120 for O3       .      104.6 Degree
PLAT398_ALERT_2_G Deviating  C-O-C   Angle From 120 for O7       .      104.5 Degree
PLAT398_ALERT_2_G Deviating  C-O-C   Angle From 120 for O11      .      103.6 Degree
PLAT606_ALERT_4_G Solvent Accessible VOID(S) in Structure .......           ! Info
PLAT794_ALERT_5_G Tentative Bond Valency for V1        (V)       .       5.01 Info
PLAT794_ALERT_5_G Tentative Bond Valency for V2        (V)       .       5.08 Info
PLAT794_ALERT_5_G Tentative Bond Valency for V3        (V)       .       5.10 Info
PLAT795_ALERT_4_G C-Atom in CIF Coordinate List Out-of-Sequence ..        C52 Note
PLAT796_ALERT_4_G O-Atom in CIF Coordinate List Out-of-Sequence ..        O10 Note
PLAT797_ALERT_4_G N-Atom in CIF Coordinate List Out-of-Sequence ..         N5 Note
PLAT868_ALERT_4_G ALERTS Due to the Use of _smtbx_masks Suppressed          ! Info
PLAT912_ALERT_4_G Missing # of FCF Reflections Above STh/L=  0.600        466 Note
PLAT933_ALERT_2_G Number of HKL-OMIT Records in Embedded .res File          8 Note
                0  3  1,   5  0  1,  -2 -1  1,  -1 -3  1,   5 -1  2,  -3  4  0,
                3 -6  1,   3 -4  1,
PLAT941_ALERT_3_G Average HKL Measurement Multiplicity ..........         1.9 Low
PLAT969_ALERT_5_G The 'Henn et al.' R-Factor-gap value ..........        2.92 Note
              Predicted wR2: Based on SigI**2  6.06 or SHELX Weight 19.12
PLAT978_ALERT_2_G Number C-C Bonds with Positive Residual Density.          0 Info
```

---

0 **ALERT level A** = Most likely a serious problem - resolve or explain
1 **ALERT level B** = A potentially serious problem, consider carefully
7 **ALERT level C** = Check. Ensure it is not caused by an omission or oversight
19 **ALERT level G** = General information/check it is not something unexpected

5 ALERT type 1 CIF construction/syntax error, inconsistent or missing data
7 ALERT type 2 Indicator that the structure model may be wrong or deficient
5 ALERT type 3 Indicator that the structure quality may be low
6 ALERT type 4 Improvement, methodology, query or suggestion
4 ALERT type 5 Informative message, check

---

checkCIF publication errors

Alert level A

```
PUBL002_ALERT_1_A  The contact author's address is missing,
           _publ_contact_author_address.
PUBL006_ALERT_1_A  _publ_requested_journal is missing
           e.g. 'Acta Crystallographica Section C'
PUBL008_ALERT_1_A  _publ_section_title is missing. Title of paper.
PUBL009_ALERT_1_A  _publ_author_name is missing. List of author(s) name(s).
PUBL010_ALERT_1_A  _publ_author_address is missing. Author(s) address(es).
PUBL012_ALERT_1_A  _publ_section_abstract is missing.
           Abstract of paper in English.
```

```
6 ALERT level A = Data missing that is essential or data in wrong format
0 ALERT level G = General alerts. Data that may be required is missing
```

Publication of your CIF

You should attempt to resolve as many as possible of the alerts in all categories. Often the minor alerts point to easily fixed oversights, errors and omissions in your CIF or refinement strategy, so attention to these fine details can be worthwhile. In order to resolve some of the more serious problems it may be necessary to carry out additional measurements or structure refinements. However, the nature of your study may justify the reported deviations from journal submission requirements and the more serious of these should be commented upon in the discussion or experimental section of a paper or in the "special_details" fields of the CIF. *checkCIF* was carefully designed to identify outliers and unusual parameters, but every test has its limitations and alerts that are not important in a particular case may appear. Conversely, the absence of alerts does not guarantee there are no aspects of the results needing attention. It is up to the individual to critically assess their own results and, if necessary, seek expert advice.

If level A alerts remain, which you believe to be justified deviations, and you intend to submit this CIF for publication in a journal, you should additionally insert an explanation in your CIF using the Validation Reply Form (VRF) below. This will allow your explanation to be considered as part of the review process.

Validation response form

Please find below a validation response form (VRF) that can be filled in and pasted into your CIF.

```
# start Validation Reply Form
_vrf_PUBL002_GLOBAL
;
PROBLEM: The contact author’s address is missing,
RESPONSE: ...
;
_vrf_PUBL006_GLOBAL
;
PROBLEM: _publ_requested_journal is missing
RESPONSE: ...
;
_vrf_PUBL008_GLOBAL
;
PROBLEM: _publ_section_title is missing. Title of paper.
RESPONSE: ...
;
_vrf_PUBL009_GLOBAL
;
PROBLEM: _publ_author_name is missing. List of author(s) name(s).
RESPONSE: ...
;
_vrf_PUBL010_GLOBAL
;
PROBLEM: _publ_author_address is missing. Author(s) address(es).
RESPONSE: ...
```

```
;
_vrf_PUBL012_GLOBAL
;
PROBLEM: _publ_section_abstract is missing.
RESPONSE: ...
;
# end Validation Reply Form
```

If you wish to submit your CIF for publication in Acta Crystallographica Section C or E, you should upload your CIF via the web. If you wish to submit your CIF for publication in IUCrData you should upload your CIF via the web. If your CIF is to form part of a submission to another IUCr journal, you will be asked, either during electronic submission or by the Co-editor handling your paper, to upload your CIF via our web site.

PLATON version of 06/01/2024; check.def file version of 05/01/2024

Datablock VO3L5-H3 - ellipsoid plot

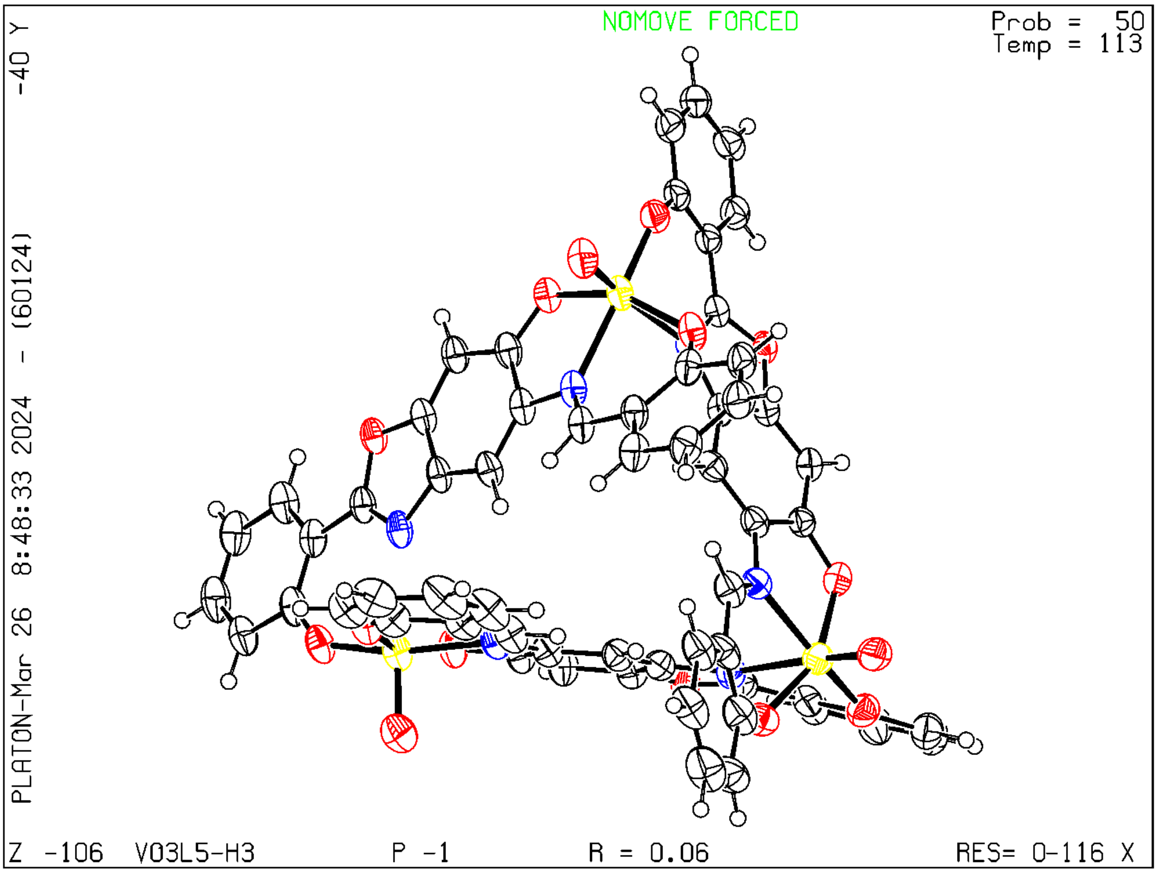

Complex 2

checkCIF/PLATON report

Structure factors have been supplied for datablock(s) VO_L5-Me_

THIS REPORT IS FOR GUIDANCE ONLY. IF USED AS PART OF A REVIEW PROCEDURE FOR PUBLICATION, IT SHOULD NOT REPLACE THE EXPERTISE OF AN EXPERIENCED CRYSTALLOGRAPHIC REFEREE.

No syntax errors found. CIF dictionary Interpreting this report

Datablock: VO_L5-Me_

Bond precision: C-C = 0.0189 A Wavelength=0.71069

Cell:	a=15.307(5)	b=15.582(5)	c=18.458(5)
	alpha=87.11(2)	beta=69.06(2)	gamma=79.74(3)
Temperature:	292 K		

	Calculated	Reported
Volume	4046(2)	4045(2)
Space group	P -1	P -1
Hall group	-P 1	-P 1
Moiety formula	C66 H45 N6 O15 V3, 2(C2 H3 N), 2(O) [+ solvent]	C70 H48 N8 O19 V3
Sum formula	C70 H51 N8 O17 V3 [+ solvent]	C70 H48 N8 O19 V3
Mr	1429.01	1458.02
Dx,g cm-3	1.173	1.197
Z	2	2
Mu (mm-1)	0.403	0.406
F000	1464.0	1490.0
F000'	1466.48	
h,k,lmax	18,18,21	18,16,21
Nref	14216	10895
Tmin,Tmax	0.864,0.960	0.885,0.960
Tmin'	0.694	

Correction method= # Reported T Limits: Tmin=0.885 Tmax=0.960
AbsCorr = PSI-SCAN

Data completeness= 0.766 Theta(max)= 24.985

R(reflections)= 0.0858(3661) wR2(reflections)= 0.3156(10895)

S = 0.927 Npar= 863

The following ALERTS were generated. Each ALERT has the format
test-name_ALERT_alert-type_alert-level.
Click on the hyperlinks for more details of the test.

Alert level A

PLAT029_ALERT_3_A _diffrn_measured_fraction_theta_full value Low . 0.766 Why?

Author Response: Low diffraction was observed for this crystal and was especially low for high angles. Due to the presence of numerous solvent molecules within the crystals, efflorescence of the crystals occurs during the reflection measurements.

Alert level B

PLAT026_ALERT_3_B Ratio Observed / Unique Reflections (too) Low .. 34% Check

Author Response: Low diffraction was observed for this crystal and was especially low for high angles. Low diffraction is also a consequence of disorder present in some solvent molecules. The final structure was satisfactory for confirming the desired chemical information.

PLAT341_ALERT_3_B Low Bond Precision on C-C Bonds .... 0.01891 Ang.

Author Response: Due to low difraction and solvent disorder, sufficient refinement could not be achieved, making it impossible to discuss bond distances, etc. However, we utilized this data only to understand the general shape of the molecular structure.

PLAT910_ALERT_3_B Missing # of FCF Reflection(s) Below Theta(Min). 15 Note

```
0  1  0,   1 -1  0,   1  0  0,   1  1  0,  -1 -1  1,  -1  0  1,
0 -1  1,   0  0  1,   0  1  1,   1 -1  1,   1  0  1,   1  1  1,
0  0  2,   1  0  2,   1  1  2,
```

Author Response: Low diffraction was observed for this crystal and was especially low for high angles. Due to the presence of numerous solvent molecules within the crystals, efflorescence of the crystals occurs during the reflection measurements.

```
PLAT911_ALERT_3_B Missing FCF Refl Between Thmin & STh/L=    0.594       3062 Report
                0  4  0,   2-16  0,   2 -1  0,   2  4  0,   2  5  0,   3-16  0,
                4-16  0,   5-16  0,   5-15  0,   6-15  0,   7-15  0,   7-14  0,
                8-14  0,   8-13  0,   8 16  0,   9-13  0,   9-12  0,   9 16  0,
               10-12  0,  10-11  0,  10 15  0,  10 16  0,  11-11  0,  11-10  0,
               11 14  0,  11 15  0,  12-10  0,  12 -9  0,  12 13  0,  12 14  0,
               12 15  0,  13 -9  0,  13 -8  0,  13 -7  0,  13 12  0,  13 13  0,
               13 14  0,  14 -7  0,  14 -6  0,  14 -5  0,  14 10  0,  14 11  0,
               14 12  0,  15 -5  0,  15 -4  0,  15 -3  0,  15 -2  0,  15  7  0,
               15  8  0,  15  9  0,  15 10  0,  15 11  0,  16 -3  0,  16 -2  0,
               16  2  0,  16  3  0,  16  4  0,  16  5  0,  16  6  0,  16  7  0,
               16  8  0,  16  9  0, -16 -8  1, -16 -7  1, -16 -6  1, -16 -5  1,
              -16 -4  1, -16 -3  1, -16 -2  1, -16  2  1, -15-10  1, -15 -9  1,
              -15 -8  1, -15 -7  1, -15 -6  1, -15 -5  1, -15  2  1, -15  3  1,
              -15  4  1, -15  5  1, -14-12  1, -14-11  1, -14-10  1, -14 -9  1,
              -14  4  1, -14  5  1, -14  6  1, -14  7  1, -13-13  1, -13-12  1,
              -13-11  1, -13  6  1, -13  7  1, -13  8  1, -12-14  1, -12-13  1,
```

Author Response: Low diffraction was observed for this crystal and was especially low for high angles. Due to the presence of numerous solvent molecules within the crystals, efflorescence of the crystals occurs during the reflection measurements.

Alert level C

```
PLAT084_ALERT_3_C High wR2 Value (i.e. > 0.25) ..................       0.32 Report
PLAT094_ALERT_2_C Ratio of Maximum / Minimum Residual Density ....       2.09 Report
PLAT202_ALERT_3_C Isotropic non-H Atoms in Anion/Solvent .........          7 Check
             N7      C67     C68     N8      C69     C70     O17B
PLAT220_ALERT_2_C NonSolvent   Resd 1  C   Ueq(max)/Ueq(min) Range        3.3 Ratio
PLAT241_ALERT_2_C High   'MainMol' Ueq as Compared to Neighbors of        C25 Check
PLAT241_ALERT_2_C High   'MainMol' Ueq as Compared to Neighbors of        C62 Check
PLAT242_ALERT_2_C Low    'MainMol' Ueq as Compared to Neighbors of        C61 Check
PLAT244_ALERT_4_C Low    'Solvent' Ueq as Compared to Neighbors of        C68 Check
PLAT244_ALERT_4_C Low    'Solvent' Ueq as Compared to Neighbors of        C70 Check
PLAT905_ALERT_3_C Negative K value in the Analysis of Variance ...    -30.630 Report
PLAT905_ALERT_3_C Negative K value in the Analysis of Variance ...     -1.696 Report
PLAT918_ALERT_3_C Reflection(s) with I(obs) much Smaller I(calc) .          2 Check
PLAT975_ALERT_2_C Check Calcd Resid. Dens.  0.88Ang From O16B    .       0.67 eA-3
PLAT975_ALERT_2_C Check Calcd Resid. Dens.  1.05Ang From O16A    .       0.66 eA-3
PLAT975_ALERT_2_C Check Calcd Resid. Dens.  0.96Ang From O16B    .       0.65 eA-3
PLAT975_ALERT_2_C Check Calcd Resid. Dens.  0.85Ang From O16B    .       0.64 eA-3
PLAT975_ALERT_2_C Check Calcd Resid. Dens.  1.00Ang From O16C    .       0.56 eA-3
PLAT975_ALERT_2_C Check Calcd Resid. Dens.  1.08Ang From O16C    .       0.54 eA-3
PLAT975_ALERT_2_C Check Calcd Resid. Dens.  0.73Ang From O16A    .       0.54 eA-3
PLAT975_ALERT_2_C Check Calcd Resid. Dens.  1.07Ang From O16C    .       0.53 eA-3
PLAT975_ALERT_2_C Check Calcd Resid. Dens.  0.65Ang From O16C    .       0.49 eA-3
PLAT975_ALERT_2_C Check Calcd Resid. Dens.  0.83Ang From O16C    .       0.48 eA-3
```

Alert level G

```
FORMU01_ALERT_2_G  There is a discrepancy between the atom counts in the
            _chemical_formula_sum and the formula from the _atom_site* data.
```

```
            Atom count from _chemical_formula_sum:C70 H48 N8 O19 V3
            Atom count from the _atom_site data:  C70 H51 N8 O17 V3
CELLZ01_ALERT_1_G Difference between formula and atom_site contents detected.
CELLZ01_ALERT_1_G ALERT: Large difference may be due to a
            symmetry error - see SYMMG tests
           From the CIF: _cell_formula_units_Z    2
           From the CIF: _chemical_formula_sum  C70 H48 N8 O19 V3
           TEST: Compare cell contents of formula and atom_site data

           atom    Z*formula  cif sites diff
           C        140.00    140.00    0.00
           H         96.00    102.00   -6.00
           N         16.00     16.00    0.00
           O         38.00     34.00    4.00
           V          6.00      6.00    0.00
CHEMS02_ALERT_1_G  Please check that you have entered the correct
            _publ_requested_category classification of your compound;
            FI or CI or EI for inorganic; FM or CM or EM for metal-organic;
            FO or CO or EO for organic.
            From the CIF: _publ_requested_category   CHOOSE FI FM FO CI CM CO or A
            From the CIF: _chemical_formula_sum :C70 H48 N8 O19 V3
PLAT002_ALERT_2_G Number of Distance or Angle Restraints on AtSite          6 Note
PLAT041_ALERT_1_G Calc. and Reported SumFormula    Strings  Differ     Please Check
              Calc: C70 H51 N8 O17 V3
              Rep.: C70 H48 N8 O19 V3
PLAT042_ALERT_1_G Calc. and Reported MoietyFormula Strings  Differ     Please Check
              Calc: C66 H45 N6 O15 V3, 2(C2 H3 N), 2(O)
              Rep.: C70 H48 N8 O19 V3
PLAT063_ALERT_4_G Crystal Size Possibly too Large for Beam Size ..       0.90 mm
PLAT072_ALERT_2_G SHELXL First  Parameter in WGHT  Unusually Large       0.17 Report
PLAT172_ALERT_4_G The CIF-Embedded .res File Contains DFIX Records          4 Report
PLAT173_ALERT_4_G The CIF-Embedded .res File Contains DANG Records          2 Report
PLAT187_ALERT_4_G The CIF-Embedded .res File Contains RIGU Records          1 Report
PLAT300_ALERT_4_G Atom Site Occupancy of O17B       Constrained at       0.55 Check
PLAT300_ALERT_4_G Atom Site Occupancy of O16A       Constrained at        0.4 Check
PLAT300_ALERT_4_G Atom Site Occupancy of O16B       Constrained at        0.4 Check
PLAT300_ALERT_4_G Atom Site Occupancy of O16C       Constrained at        0.2 Check
PLAT300_ALERT_4_G Atom Site Occupancy of O17A       Constrained at       0.45 Check
PLAT302_ALERT_4_G Anion/Solvent/Minor-Residue Disorder (Resd    4)       100% Note
PLAT302_ALERT_4_G Anion/Solvent/Minor-Residue Disorder (Resd    5)       100% Note
PLAT302_ALERT_4_G Anion/Solvent/Minor-Residue Disorder (Resd    6)       100% Note
PLAT302_ALERT_4_G Anion/Solvent/Minor-Residue Disorder (Resd    7)       100% Note
PLAT302_ALERT_4_G Anion/Solvent/Minor-Residue Disorder (Resd    8)       100% Note
PLAT304_ALERT_4_G Non-Integer Number of Atoms in ..... (Resd    4)       0.55 Check
PLAT304_ALERT_4_G Non-Integer Number of Atoms in ..... (Resd    5)       0.40 Check
PLAT304_ALERT_4_G Non-Integer Number of Atoms in ..... (Resd    6)       0.40 Check
PLAT304_ALERT_4_G Non-Integer Number of Atoms in ..... (Resd    7)       0.20 Check
PLAT304_ALERT_4_G Non-Integer Number of Atoms in ..... (Resd    8)       0.45 Check
PLAT311_ALERT_2_G Isolated Disordered Oxygen Atom (No H's ?) .....       O17B Check
PLAT311_ALERT_2_G Isolated Disordered Oxygen Atom (No H's ?) .....       O16A Check
PLAT311_ALERT_2_G Isolated Disordered Oxygen Atom (No H's ?) .....       O16B Check
PLAT311_ALERT_2_G Isolated Disordered Oxygen Atom (No H's ?) .....       O16C Check
PLAT311_ALERT_2_G Isolated Disordered Oxygen Atom (No H's ?) .....       O17A Check
PLAT344_ALERT_2_G Unusual sp?       Angle Range in Solvent/Ion for        C70 Check
PLAT367_ALERT_2_G Long?  C(sp?)-C(sp?) Bond  C69      - C70      .       1.53 Ang.
PLAT398_ALERT_2_G Deviating  C-O-C   Angle From 120 for O3       .      105.1 Degree
PLAT398_ALERT_2_G Deviating  C-O-C   Angle From 120 for O7       .      104.3 Degree
```

```
PLAT398_ALERT_2_G Deviating  C-O-C   Angle From 120 for O11      .      103.6 Degree
PLAT606_ALERT_4_G Solvent Accessible VOID(S) in Structure ........          ! Info
PLAT790_ALERT_4_G Centre of Gravity not Within Unit Cell: Resd.  #          3 Note
              C2 H3 N
PLAT794_ALERT_5_G Tentative Bond Valency for V1        (V)       .       5.28 Info
PLAT794_ALERT_5_G Tentative Bond Valency for V2        (V)       .       5.09 Info
PLAT794_ALERT_5_G Tentative Bond Valency for V3        (V)       .       5.21 Info
PLAT860_ALERT_3_G Number of Least-Squares Restraints ............         942 Note
PLAT869_ALERT_4_G ALERTS Related to the Use of SQUEEZE  Suppressed          ! Info
PLAT908_ALERT_2_G Max. Perc. Data with I > 2*s(I) per Res.Shell .      71.97% Note
PLAT933_ALERT_2_G Number of HKL-OMIT Records in Embedded .res File         28 Note
                0 -2  1,  -3 -1  1,  -3  0  1,   3  1  2,  -1 -1  4,   4  0  1,
                3  0  2,   0  0  3,  -4  2  2,  -2  1  0,   2 -1  2,   0  4  0,
               -4  0  1,  -2  1  2,   4  1  4,   2  4  0,  -2  0  1,   0  1  3,
                1  1  2,   4  3  3,   2  0  2,   2  5  0,   3  2  2,   2  5  3,
               -1 -1  6,  -1  1  4,  -1 -5  1,  -1 -2  1,
PLAT941_ALERT_3_G Average HKL Measurement Multiplicity ..........         1.1 Low
PLAT951_ALERT_5_G Calculated (ThMax) and CIF-Reported Kmax Differ           2 Units
PLAT957_ALERT_1_G Calculated (ThMax) and Actual (FCF) Kmax Differ           2 Units
PLAT969_ALERT_5_G The 'Henn et al.' R-Factor-gap value ..........       4.18 Note
              Predicted wR2: Based on SigI**2  7.54 or SHELX Weight 35.48
PLAT978_ALERT_2_G Number C-C Bonds with Positive Residual Density.          0 Info
```

```
   1 ALERT level A = Most likely a serious problem - resolve or explain
   4 ALERT level B = A potentially serious problem, consider carefully
  22 ALERT level C = Check. Ensure it is not caused by an omission or oversight
  51 ALERT level G = General information/check it is not something unexpected

   6 ALERT type 1 CIF construction/syntax error, inconsistent or missing data
  31 ALERT type 2 Indicator that the structure model may be wrong or deficient
  12 ALERT type 3 Indicator that the structure quality may be low
  24 ALERT type 4 Improvement, methodology, query or suggestion
   5 ALERT type 5 Informative message, check
```

---

checkCIF publication errors

Alert level A

```
PUBL002_ALERT_1_A  The contact author's address is missing,
            _publ_contact_author_address.
```

```
   1 ALERT level A = Data missing that is essential or data in wrong format
   0 ALERT level G = General alerts. Data that may be required is missing
```

Publication of your CIF

You should attempt to resolve as many as possible of the alerts in all categories. Often the minor alerts point to easily fixed oversights, errors and omissions in your CIF or refinement strategy, so attention to these fine details can be worthwhile. In order to resolve some of the more serious problems it may be necessary to carry out additional measurements or structure refinements. However, the nature of your study may justify the reported deviations from journal submission requirements and the more serious of these should be commented upon in the discussion or experimental section of a paper or in the "special_details" fields of the CIF. *checkCIF* was carefully designed to identify outliers and unusual parameters, but every test has its limitations and alerts that are not important in a particular case may appear. Conversely, the absence of alerts does not guarantee there are no aspects of the results needing attention. It is up to the individual to critically assess their own results and, if necessary, seek expert advice.

If level A alerts remain, which you believe to be justified deviations, and you intend to submit this CIF for publication in a journal, you should additionally insert an explanation in your CIF using the Validation Reply Form (VRF) below. This will allow your explanation to be considered as part of the review process.

Validation response form

Please find below a validation response form (VRF) that can be filled in and pasted into your CIF.

```
# start Validation Reply Form
_vrf_PUBL002_GLOBAL
;
PROBLEM: The contact author's address is missing,
RESPONSE: ...
;
# end Validation Reply Form
```

If you wish to submit your CIF for publication in Acta Crystallographica Section C or E, you should upload your CIF via the web. If you wish to submit your CIF for publication in IUCrData you should upload your CIF via the web. If your CIF is to form part of a submission to another IUCr journal, you will be asked, either during electronic submission or by the Co-editor handling your paper, to upload your CIF via our web site.

PLATON version of 06/01/2024; check.def file version of 05/01/2024

Datablock VO_L5-Me_ - ellipsoid plot

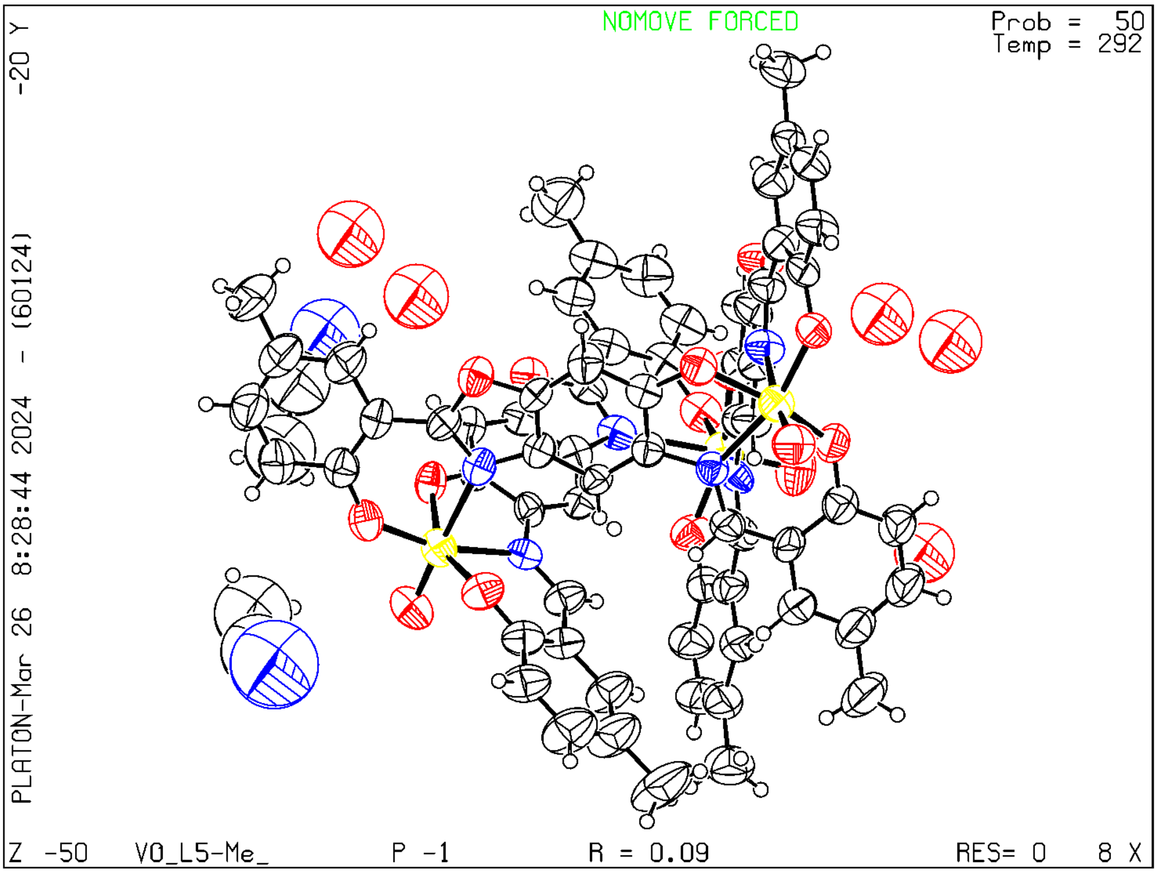

Complex 4

checkCIF/PLATON report

Structure factors have been supplied for datablock(s) HNEt3VO2L5-H

THIS REPORT IS FOR GUIDANCE ONLY. IF USED AS PART OF A REVIEW PROCEDURE FOR PUBLICATION, IT SHOULD NOT REPLACE THE EXPERTISE OF AN EXPERIENCED CRYSTALLOGRAPHIC REFEREE.

No syntax errors found. CIF dictionary Interpreting this report

Datablock: HNEt3VO2L5-H

Bond precision:	C-C = 0.0124 A		Wavelength=0.71069
Cell:	a=11.978(3)	b=7.5880(14)	c=28.042(5)
	alpha=90	beta=101.705(18)	gamma=90
Temperature:	301 K		

	Calculated	Reported
Volume	2495.7(9)	2495.7(8)
Space group	P 21/n	P 1 21/n 1
Hall group	-P 2yn	-P 2yn
Moiety formula	C20 H12 N2 O6 V, C6 H16 N	C26 H28 N3 O6 V
Sum formula	C26 H28 N3 O6 V	C26 H28 N3 O6 V
Mr	529.45	529.47
Dx,g cm-3	1.409	1.409
Z	4	4
Mu (mm-1)	0.444	0.444
F000	1104.0	1104.0
F000’	1105.69	
h,k,lmax	15,9,36	15,9,36
Nref	5734	5478
Tmin,Tmax	0.899,0.957	
Tmin’	0.837	

Correction method= Not given

Data completeness= 0.955 Theta(max)= 27.499

R(reflections)= 0.0790(1828) wR2(reflections)= 0.3181(5478)

S = 0.947 Npar= 329

The following ALERTS were generated. Each ALERT has the format
test-name_ALERT_alert-type_alert-level.
Click on the hyperlinks for more details of the test.

Alert level B

```
PLAT026_ALERT_3_B Ratio Observed / Unique Reflections (too) Low ..        33% Check
```

Author Response: Low diffraction was observed for this crystal and was especially low for high angles.

Alert level C

```
PLAT042_ALERT_1_C Calc. and Reported MoietyFormula Strings  Differ     Please Check
              Calc: C20 H12 N2 O6 V, C6 H16 N
              Rep.: C26 H28 N3 O6 V
PLAT084_ALERT_3_C High wR2 Value (i.e. > 0.25) ...................       0.32 Report
PLAT234_ALERT_4_C Large Hirshfeld Difference C25      --C26      .       0.24 Ang.
PLAT243_ALERT_4_C High   'Solvent' Ueq as Compared to Neighbors of        C25 Check
PLAT244_ALERT_4_C Low    'Solvent' Ueq as Compared to Neighbors of         N3 Check
PLAT244_ALERT_4_C Low    'Solvent' Ueq as Compared to Neighbors of        C21 Check
PLAT245_ALERT_2_C U(iso) H12       Smaller than U(eq) O4        by      0.028 Ang**2
PLAT260_ALERT_2_C Large Average Ueq of Residue Including        N3      0.137 Check
PLAT341_ALERT_3_C Low Bond Precision on  C-C Bonds ..............    0.01243 Ang.
PLAT360_ALERT_2_C Short  C(sp3)-C(sp3) Bond  C21       - C22      .       1.42 Ang.
PLAT360_ALERT_2_C Short  C(sp3)-C(sp3) Bond  C25       - C26      .       1.39 Ang.
PLAT745_ALERT_1_C D-H     Calc     0.77(8), Rep     0.77100 ......    Missing s.u.
              O4       -H12             1_555   1_555 ........     #    1 Check
PLAT746_ALERT_1_C H...A   Calc     1.98(8), Rep     1.97600 ......    Missing s.u.
              H12      -N2              1_555   1_555 ........     #    1 Check
PLAT748_ALERT_1_C D-H..A  Calc      148(7), Rep      148.20 ......    Missing s.u.
              O4      -H12     -N2         1_555  1_555  1_555     #    1 Check
PLAT905_ALERT_3_C Negative K value in the Analysis of Variance ...     -0.125 Report
PLAT906_ALERT_3_C Large K Value in the Analysis of Variance ......      6.440 Check
PLAT911_ALERT_3_C Missing FCF Refl Between Thmin & STh/L=    0.600         50 Report
                0  2  0,   2  0  0,   2  1  0,   2  2  0, -14  1  1,   0  2  1,
                1  1  1,   3  2  1,   5  0  1,  -2  0  2,  -1  1  2,  -1  2  2,
                0  1  2,   2  1  2,   2  3  2,  -5  0  3,  -3  0  3,  -2  1  3,
               -1  1  3,  -1  2  3,   2  1  3,   3  0  3,  -2  2  4,  -1  1  4,
               -1  2  4,   0  2  4, -14  2  5, -12  5  5,  -5  0  5,  -1  0  5,
               -1  1  5, -14  2  6, -12  5  6, -14  2  7, -14  2  8,  -6  4  9,
               -2  2  9,  -3  3 11, -14  1 12, -14  1 13,  -3  0 13, -14  0 14,
              -13  3 14, -13  3 15,  -1  0 15, -12  4 16, -13  2 18, -12  3 20,
              -12  2 22, -12  1 23,
PLAT913_ALERT_3_C Missing # of Very Strong Reflections in FCF ....          4 Note
                2  0  0,  -1  2  2,  -1  2  3,  -1  2  4,
```

Alert level G

```
CHEMS02_ALERT_1_G  Please check that you have entered the correct
            _publ_requested_category classification of your compound;
            FI or CI or EI for inorganic; FM or CM or EM for metal-organic;
            FO or CO or EO for organic.
```

```
           From the CIF: _publ_requested_category   CHOOSE FI FM FO CI CM CO or A
           From the CIF: _chemical_formula_sum :C26 H28 N3 O6 V1
PLAT003_ALERT_2_G Number of Uiso or Uij Restrained non-H Atoms ...         36 Report
PLAT007_ALERT_5_G Number of Unrefined Donor-H Atoms .............           1 Report
             H28
PLAT072_ALERT_2_G SHELXL First  Parameter in WGHT  Unusually Large       0.16 Report
PLAT177_ALERT_4_G The CIF-Embedded .res File Contains DELU Records           1 Report
PLAT398_ALERT_2_G Deviating  C-O-C   Angle From 120 for O3       .      105.2 Degree
PLAT484_ALERT_4_G Round D-H..A Angle Rep for O4       ..N2       .        148 Degree
PLAT484_ALERT_4_G Round D-H..A Angle Rep for N3       ..O5       .        156 Degree
PLAT484_ALERT_4_G Round D-H..A Angle Rep for N3       ..O6       .        128 Degree
PLAT794_ALERT_5_G Tentative Bond Valency for V1        (V)       .       5.12 Info
PLAT860_ALERT_3_G Number of Least-Squares Restraints ............         101 Note
PLAT910_ALERT_3_G Missing # of FCF Reflection(s) Below Theta(Min).          3 Note
              -1  0  1,   1  0  1,   0  0  2,
PLAT912_ALERT_4_G Missing # of FCF Reflections Above STh/L=  0.600        203 Note
PLAT933_ALERT_2_G Number of HKL-OMIT Records in Embedded .res File         35 Note
              -1  2  3,  -1  2  2,  -1  0  5,  -5  0  3,   2  2  0,   0  1  2,
              -1  1  2,   3  0  3,  -1  2  4,   2  1  2,   2  1  0,   2  2  0,
              -1  1  4,  -1  1  5,  -2  1  3,   2  3  2,   2  1  3,  -1  0 15,
               0  2  0,  -3  0 13,  -2  2  4,  -2  0  2,   2  1  0,   5  0  1,
              -3  3 11,  -5  0  5,   3  2  1,   0  2  1,  -1  1  3,  -3  0  3,
               0  2  4,   1  1  1,   2  0  0,   2  0  0,  -2  2  9,
PLAT941_ALERT_3_G Average HKL Measurement Multiplicity ..........         1.0 Low
PLAT969_ALERT_5_G The 'Henn et al.' R-Factor-gap value ..........        2.34 Note
             Predicted wR2: Based on SigI**2 13.58 or SHELX Weight 34.65
PLAT978_ALERT_2_G Number C-C Bonds with Positive Residual Density.          0 Info
```

---

0 **ALERT level A** = Most likely a serious problem - resolve or explain
1 **ALERT level B** = A potentially serious problem, consider carefully
18 **ALERT level C** = Check. Ensure it is not caused by an omission or oversight
17 **ALERT level G** = General information/check it is not something unexpected

5 ALERT type 1 CIF construction/syntax error, inconsistent or missing data
9 ALERT type 2 Indicator that the structure model may be wrong or deficient
10 ALERT type 3 Indicator that the structure quality may be low
9 ALERT type 4 Improvement, methodology, query or suggestion
3 ALERT type 5 Informative message, check

---

checkCIF publication errors

Alert level A

PUBL002_ALERT_1_A The contact author's address is missing,
_publ_contact_author_address.

---

1 **ALERT level A** = Data missing that is essential or data in wrong format
0 **ALERT level G** = General alerts. Data that may be required is missing

---

Publication of your CIF

You should attempt to resolve as many as possible of the alerts in all categories. Often the minor alerts point to easily fixed oversights, errors and omissions in your CIF or refinement strategy, so attention to these fine details can be worthwhile. In order to resolve some of the more serious problems it may be necessary to carry out additional measurements or structure refinements. However, the nature of your study may justify the reported deviations from journal submission requirements and the more serious of these should be commented upon in the discussion or experimental section of a paper or in the "special_details" fields of the CIF. *checkCIF* was carefully designed to identify outliers and unusual parameters, but every test has its limitations and alerts that are not important in a particular case may appear. Conversely, the absence of alerts does not guarantee there are no aspects of the results needing attention. It is up to the individual to critically assess their own results and, if necessary, seek expert advice.

If level A alerts remain, which you believe to be justified deviations, and you intend to submit this CIF for publication in a journal, you should additionally insert an explanation in your CIF using the Validation Reply Form (VRF) below. This will allow your explanation to be considered as part of the review process.

Validation response form

Please find below a validation response form (VRF) that can be filled in and pasted into your CIF.

```
# start Validation Reply Form
_vrf_PUBL002_GLOBAL
;
PROBLEM: The contact author's address is missing,
RESPONSE: ...
;
# end Validation Reply Form
```

If you wish to submit your CIF for publication in Acta Crystallographica Section C or E, you should upload your CIF via the web. If you wish to submit your CIF for publication in IUCrData you should upload your CIF via the web. If your CIF is to form part of a submission to another IUCr journal, you will be asked, either during electronic submission or by the Co-editor handling your paper, to upload your CIF via our web site.

PLATON version of 06/01/2024; check.def file version of 05/01/2024

Datablock HNEt3VO2L5-H - ellipsoid plot

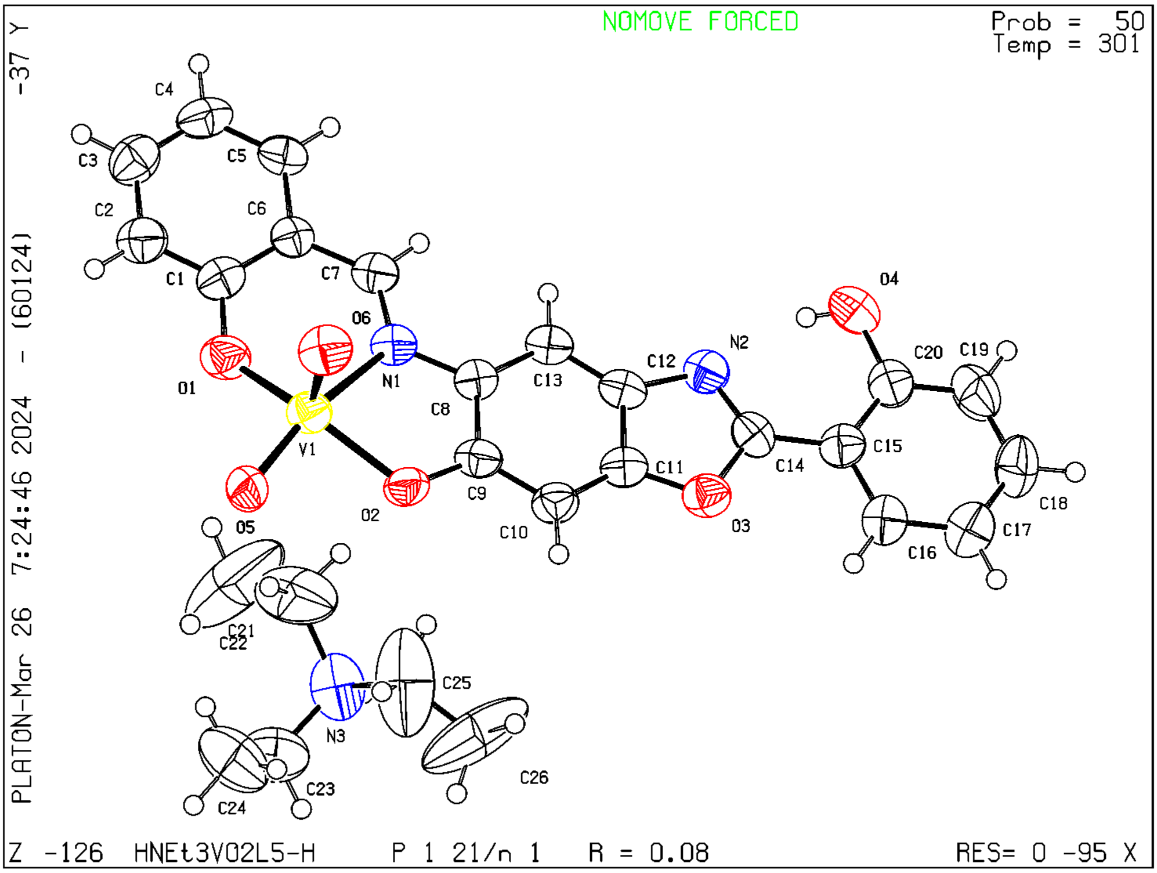

Supplement: Supplementary file 1 [file molecules-29-01700-s001.zip › SupplementaryMaterials_Rev1.pdf]
